# Supplementary material for: Genomic surveillance of SARS-CoV-2 in Puerto Rico enabled early detection and tracking of variants
Source: Commun Med (Lond). 2022 Aug 11;2:100. doi: 10.1038/s43856-022-00168-7 (PMC9366129; doi:10.1038/s43856-022-00168-7)
Supplement: Supplementary file 1 — Description of Additional Supplementary Files [file 43856_2022_168_MOESM1_ESM.pdf]

Description of Additional Supplementary Files

File Name: Supplementary Data 1

Description: List of genome sequences from Puerto Rico generated by this study.
