# Supplementary material for: Genomic surveillance of SARS-CoV-2 in Puerto Rico enabled early detection and tracking of variants
Source: Commun Med (Lond). 2022 Aug 11;2:100. doi: 10.1038/s43856-022-00168-7 (PMC9366129; doi:10.1038/s43856-022-00168-7)
Supplement: Supplementary file 2 — Supplementary Data 1 [file 43856_2022_168_MOESM2_ESM.docx]

**Supplementary Data 1. Genomes generated by this study**

| Accession ID | Virus Name | Location | Collection Date |
| --- | --- | --- | --- |
| EPI_ISL_1120708 | hCoV-19/Puerto Rico/PR-CDC-S168/2021 | North America / Puerto Rico | 2021-01-22 |
| EPI_ISL_1168653 | hCoV-19/Puerto Rico/PR-CDC-S128/2020 | North America / Puerto Rico | 2020-11-16 |
| EPI_ISL_1168654 | hCoV-19/Puerto Rico/PR-CDC-S129/2020 | North America / Puerto Rico | 2020-12-06 |
| EPI_ISL_1168655 | hCoV-19/Puerto Rico/PR-CDC-S130/2021 | North America / Puerto Rico | 2021-01-21 |
| EPI_ISL_1168656 | hCoV-19/Puerto Rico/PR-CDC-S131/2020 | North America / Puerto Rico | 2020-12-21 |
| EPI_ISL_1168657 | hCoV-19/Puerto Rico/PR-CDC-S132/2021 | North America / Puerto Rico | 2021-01-21 |
| EPI_ISL_1168658 | hCoV-19/Puerto Rico/PR-CDC-S133/2020 | North America / Puerto Rico | 2020-12-19 |
| EPI_ISL_1168659 | hCoV-19/Puerto Rico/PR-CDC-S134/2020 | North America / Puerto Rico | 2020-12-19 |
| EPI_ISL_1168660 | hCoV-19/Puerto Rico/PR-CDC-S135/2020 | North America / Puerto Rico | 2020-10-28 |
| EPI_ISL_1168661 | hCoV-19/Puerto Rico/PR-CDC-S136/2020 | North America / Puerto Rico | 2020-10-30 |
| EPI_ISL_1168662 | hCoV-19/Puerto Rico/PR-CDC-S137/2020 | North America / Puerto Rico | 2020-11-19 |
| EPI_ISL_1168663 | hCoV-19/Puerto Rico/PR-CDC-S138/2020 | North America / Puerto Rico | 2020-11-13 |
| EPI_ISL_1168664 | hCoV-19/Puerto Rico/PR-CDC-S139/2020 | North America / Puerto Rico | 2020-11-23 |
| EPI_ISL_1168665 | hCoV-19/Puerto Rico/PR-CDC-S140/2020 | North America / Puerto Rico | 2020-12-14 |
| EPI_ISL_1168666 | hCoV-19/Puerto Rico/PR-CDC-S141/2020 | North America / Puerto Rico | 2020-12-17 |
| EPI_ISL_1168667 | hCoV-19/Puerto Rico/PR-CDC-S142/2021 | North America / Puerto Rico | 2021-01-14 |
| EPI_ISL_1168668 | hCoV-19/Puerto Rico/PR-CDC-S143/2020 | North America / Puerto Rico | 2020-11-05 |
| EPI_ISL_1168669 | hCoV-19/Puerto Rico/PR-CDC-S144/2020 | North America / Puerto Rico | 2020-11-12 |
| EPI_ISL_1168670 | hCoV-19/Puerto Rico/PR-CDC-S145/2020 | North America / Puerto Rico | 2020-11-12 |
| EPI_ISL_1168671 | hCoV-19/Puerto Rico/PR-CDC-S146/2020 | North America / Puerto Rico | 2020-10-26 |
| EPI_ISL_1168672 | hCoV-19/Puerto Rico/PR-CDC-S147/2020 | North America / Puerto Rico | 2020-12-16 |
| EPI_ISL_1168673 | hCoV-19/Puerto Rico/PR-CDC-S148/2020 | North America / Puerto Rico | 2020-12-02 |
| EPI_ISL_1168674 | hCoV-19/Puerto Rico/PR-CDC-S149/2021 | North America / Puerto Rico | 2021-01-07 |
| EPI_ISL_1168675 | hCoV-19/Puerto Rico/PR-CDC-S150/2020 | North America / Puerto Rico | 2020-12-01 |
| EPI_ISL_1168676 | hCoV-19/Puerto Rico/PR-CDC-S151/2021 | North America / Puerto Rico | 2021-01-21 |
| EPI_ISL_1168677 | hCoV-19/Puerto Rico/PR-CDC-S152/2020 | North America / Puerto Rico | 2020-10-30 |
| EPI_ISL_1168678 | hCoV-19/Puerto Rico/PR-CDC-S153/2020 | North America / Puerto Rico | 2020-11-02 |
| EPI_ISL_1168679 | hCoV-19/Puerto Rico/PR-CDC-S154/2020 | North America / Puerto Rico | 2020-11-07 |
| EPI_ISL_1168680 | hCoV-19/Puerto Rico/PR-CDC-S155/2020 | North America / Puerto Rico | 2020-12-08 |
| EPI_ISL_1168681 | hCoV-19/Puerto Rico/PR-CDC-S156/2020 | North America / Puerto Rico | 2020-12-14 |
| EPI_ISL_1168682 | hCoV-19/Puerto Rico/PR-CDC-S157/2020 | North America / Puerto Rico | 2020-11-18 |
| EPI_ISL_1168683 | hCoV-19/Puerto Rico/PR-CDC-S158/2021 | North America / Puerto Rico | 2021-01-18 |
| EPI_ISL_1168684 | hCoV-19/Puerto Rico/PR-CDC-S159/2021 | North America / Puerto Rico | 2021-01-18 |
| EPI_ISL_1168685 | hCoV-19/Puerto Rico/PR-CDC-S160/2021 | North America / Puerto Rico | 2021-01-26 |
| EPI_ISL_1168686 | hCoV-19/Puerto Rico/PR-CDC-S161/2021 | North America / Puerto Rico | 2021-01-12 |
| EPI_ISL_1168687 | hCoV-19/Puerto Rico/PR-CDC-S162/2021 | North America / Puerto Rico | 2021-01-08 |
| EPI_ISL_1168688 | hCoV-19/Puerto Rico/PR-CDC-S163/2021 | North America / Puerto Rico | 2021-01-14 |
| EPI_ISL_1168689 | hCoV-19/Puerto Rico/PR-CDC-S164/2020 | North America / Puerto Rico | 2020-12-09 |
| EPI_ISL_1168690 | hCoV-19/Puerto Rico/PR-CDC-S165/2021 | North America / Puerto Rico | 2021-01-12 |
| EPI_ISL_1168691 | hCoV-19/Puerto Rico/PR-CDC-S166/2021 | North America / Puerto Rico | 2021-01-12 |
| EPI_ISL_1168692 | hCoV-19/Puerto Rico/PR-CDC-S167/2021 | North America / Puerto Rico | 2021-01-12 |
| EPI_ISL_1168693 | hCoV-19/Puerto Rico/PR-CDC-S172/2020 | North America / Puerto Rico | 2020-08-02 |
| EPI_ISL_1168694 | hCoV-19/Puerto Rico/PR-CDC-S169/2020 | North America / Puerto Rico | 2020-07-07 |
| EPI_ISL_1168695 | hCoV-19/Puerto Rico/PR-CDC-S170/2020 | North America / Puerto Rico | 2020-08-04 |
| EPI_ISL_1168696 | hCoV-19/Puerto Rico/PR-CDC-S171/2020 | North America / Puerto Rico | 2020-11-24 |
| EPI_ISL_1620533 | hCoV-19/Puerto Rico/PR-CDC-S240/2021 | North America / Puerto Rico | 2021-03-20 |
| EPI_ISL_1620534 | hCoV-19/Puerto Rico/PR-CDC-S226/2021 | North America / Puerto Rico | 2021-03-10 |
| EPI_ISL_1620535 | hCoV-19/Puerto Rico/PR-CDC-S212/2021 | North America / Puerto Rico | 2021-03-10 |
| EPI_ISL_1620536 | hCoV-19/Puerto Rico/PR-CDC-S173/2021 | North America / Puerto Rico | 2021-03-02 |
| EPI_ISL_1620537 | hCoV-19/Puerto Rico/PR-CDC-S210/2021 | North America / Puerto Rico | 2021-03-11 |
| EPI_ISL_1620538 | hCoV-19/Puerto Rico/PR-CDC-S221/2021 | North America / Puerto Rico | 2021-03-10 |
| EPI_ISL_1620539 | hCoV-19/Puerto Rico/PR-CDC-S222/2021 | North America / Puerto Rico | 2021-03-10 |
| EPI_ISL_1620540 | hCoV-19/Puerto Rico/PR-CDC-S223/2021 | North America / Puerto Rico | 2021-03-09 |
| EPI_ISL_1620541 | hCoV-19/Puerto Rico/PR-CDC-S224/2021 | North America / Puerto Rico | 2021-03-09 |
| EPI_ISL_1620542 | hCoV-19/Puerto Rico/PR-CDC-S225/2021 | North America / Puerto Rico | 2021-03-09 |
| EPI_ISL_1620543 | hCoV-19/Puerto Rico/PR-CDC-S227/2021 | North America / Puerto Rico | 2021-03-12 |
| EPI_ISL_1620544 | hCoV-19/Puerto Rico/PR-CDC-S246/2021 | North America / Puerto Rico | 2021-03-18 |
| EPI_ISL_1620545 | hCoV-19/Puerto Rico/PR-CDC-S247/2021 | North America / Puerto Rico | 2021-03-18 |
| EPI_ISL_1620546 | hCoV-19/Puerto Rico/PR-CDC-S175/2021 | North America / Puerto Rico | 2021-03-03 |
| EPI_ISL_1620547 | hCoV-19/Puerto Rico/PR-CDC-S180/2021 | North America / Puerto Rico | 2021-03-04 |
| EPI_ISL_1620548 | hCoV-19/Puerto Rico/PR-CDC-S183/2021 | North America / Puerto Rico | 2021-03-05 |
| EPI_ISL_1620549 | hCoV-19/Puerto Rico/PR-CDC-S195/2021 | North America / Puerto Rico | 2021-03-08 |
| EPI_ISL_1620550 | hCoV-19/Puerto Rico/PR-CDC-S203/2021 | North America / Puerto Rico | 2021-03-08 |
| EPI_ISL_1620551 | hCoV-19/Puerto Rico/PR-CDC-S208/2021 | North America / Puerto Rico | 2021-03-10 |
| EPI_ISL_1620552 | hCoV-19/Puerto Rico/PR-CDC-S200/2021 | North America / Puerto Rico | 2021-03-08 |
| EPI_ISL_1620553 | hCoV-19/Puerto Rico/PR-CDC-S213/2021 | North America / Puerto Rico | 2021-03-10 |
| EPI_ISL_1620554 | hCoV-19/Puerto Rico/PR-CDC-S217/2021 | North America / Puerto Rico | 2021-03-18 |
| EPI_ISL_1620555 | hCoV-19/Puerto Rico/PR-CDC-S219/2021 | North America / Puerto Rico | 2021-03-04 |
| EPI_ISL_1620556 | hCoV-19/Puerto Rico/PR-CDC-S228/2021 | North America / Puerto Rico | 2021-03-15 |
| EPI_ISL_1620557 | hCoV-19/Puerto Rico/PR-CDC-S229/2021 | North America / Puerto Rico | 2021-03-15 |
| EPI_ISL_1620558 | hCoV-19/Puerto Rico/PR-CDC-S230/2021 | North America / Puerto Rico | 2021-03-15 |
| EPI_ISL_1620559 | hCoV-19/Puerto Rico/PR-CDC-S231/2021 | North America / Puerto Rico | 2021-03-17 |
| EPI_ISL_1620560 | hCoV-19/Puerto Rico/PR-CDC-S233/2021 | North America / Puerto Rico | 2021-03-18 |
| EPI_ISL_1620561 | hCoV-19/Puerto Rico/PR-CDC-S234/2021 | North America / Puerto Rico | 2021-03-18 |
| EPI_ISL_1620562 | hCoV-19/Puerto Rico/PR-CDC-S235/2021 | North America / Puerto Rico | 2021-03-19 |
| EPI_ISL_1620563 | hCoV-19/Puerto Rico/PR-CDC-S236/2021 | North America / Puerto Rico | 2021-03-19 |
| EPI_ISL_1620564 | hCoV-19/Puerto Rico/PR-CDC-S237/2021 | North America / Puerto Rico | 2021-03-19 |
| EPI_ISL_1620565 | hCoV-19/Puerto Rico/PR-CDC-S238/2021 | North America / Puerto Rico | 2021-03-18 |
| EPI_ISL_1620566 | hCoV-19/Puerto Rico/PR-CDC-S242/2021 | North America / Puerto Rico | 2021-03-16 |
| EPI_ISL_1620567 | hCoV-19/Puerto Rico/PR-CDC-S243/2021 | North America / Puerto Rico | 2021-03-22 |
| EPI_ISL_1620568 | hCoV-19/Puerto Rico/PR-CDC-S244/2021 | North America / Puerto Rico | 2021-03-22 |
| EPI_ISL_1620569 | hCoV-19/Puerto Rico/PR-CDC-S248/2021 | North America / Puerto Rico | 2021-03-24 |
| EPI_ISL_1620570 | hCoV-19/Puerto Rico/PR-CDC-S251/2021 | North America / Puerto Rico | 2021-03-23 |
| EPI_ISL_1620571 | hCoV-19/Puerto Rico/PR-CDC-S255/2021 | North America / Puerto Rico | 2021-03-24 |
| EPI_ISL_1620572 | hCoV-19/Puerto Rico/PR-CDC-S256/2021 | North America / Puerto Rico | 2021-03-23 |
| EPI_ISL_1620573 | hCoV-19/Puerto Rico/PR-CDC-S258/2021 | North America / Puerto Rico | 1905-07-13 |
| EPI_ISL_1620574 | hCoV-19/Puerto Rico/PR-CDC-S262/2021 | North America / Puerto Rico | 2021-03-30 |
| EPI_ISL_1620575 | hCoV-19/Puerto Rico/PR-CDC-S263/2021 | North America / Puerto Rico | 2021-03-30 |
| EPI_ISL_1620576 | hCoV-19/Puerto Rico/PR-CDC-S264/2021 | North America / Puerto Rico | 2021-03-30 |
| EPI_ISL_1620577 | hCoV-19/Puerto Rico/PR-CDC-S265/2021 | North America / Puerto Rico | 2021-03-30 |
| EPI_ISL_1620578 | hCoV-19/Puerto Rico/PR-CDC-S266/2021 | North America / Puerto Rico | 2021-03-30 |
| EPI_ISL_1620579 | hCoV-19/Puerto Rico/PR-CDC-S267/2021 | North America / Puerto Rico | 2021-03-30 |
| EPI_ISL_1620580 | hCoV-19/Puerto Rico/PR-CDC-S186/2021 | North America / Puerto Rico | 2021-03-06 |
| EPI_ISL_1620581 | hCoV-19/Puerto Rico/PR-CDC-S220/2021 | North America / Puerto Rico | 2021-03-08 |
| EPI_ISL_1620582 | hCoV-19/Puerto Rico/PR-CDC-S232/2021 | North America / Puerto Rico | 2021-03-17 |
| EPI_ISL_1620583 | hCoV-19/Puerto Rico/PR-CDC-S239/2021 | North America / Puerto Rico | 2021-03-19 |
| EPI_ISL_1620584 | hCoV-19/Puerto Rico/PR-CDC-S241/2021 | North America / Puerto Rico | 2021-03-22 |
| EPI_ISL_1620585 | hCoV-19/Puerto Rico/PR-CDC-S249/2021 | North America / Puerto Rico | 2021-03-23 |
| EPI_ISL_1620586 | hCoV-19/Puerto Rico/PR-CDC-S250/2021 | North America / Puerto Rico | 2021-03-23 |
| EPI_ISL_1620587 | hCoV-19/Puerto Rico/PR-CDC-S252/2021 | North America / Puerto Rico | 2021-03-23 |
| EPI_ISL_1620588 | hCoV-19/Puerto Rico/PR-CDC-S253/2021 | North America / Puerto Rico | 2021-03-23 |
| EPI_ISL_1620589 | hCoV-19/Puerto Rico/PR-CDC-S254/2021 | North America / Puerto Rico | 2021-03-23 |
| EPI_ISL_1620590 | hCoV-19/Puerto Rico/PR-CDC-S257/2021 | North America / Puerto Rico | 1905-07-13 |
| EPI_ISL_1620591 | hCoV-19/Puerto Rico/PR-CDC-S259/2021 | North America / Puerto Rico | 2021-03-29 |
| EPI_ISL_1620592 | hCoV-19/Puerto Rico/PR-CDC-S260/2021 | North America / Puerto Rico | 2021-03-24 |
| EPI_ISL_1620593 | hCoV-19/Puerto Rico/PR-CDC-S261/2021 | North America / Puerto Rico | 2021-03-30 |
| EPI_ISL_1620594 | hCoV-19/Puerto Rico/PR-CDC-S174/2021 | North America / Puerto Rico | 2021-03-02 |
| EPI_ISL_1620595 | hCoV-19/Puerto Rico/PR-CDC-S214/2021 | North America / Puerto Rico | 2021-03-11 |
| EPI_ISL_1620596 | hCoV-19/Puerto Rico/PR-CDC-S179/2021 | North America / Puerto Rico | 2021-03-03 |
| EPI_ISL_1620597 | hCoV-19/Puerto Rico/PR-CDC-S198/2021 | North America / Puerto Rico | 2021-03-08 |
| EPI_ISL_1620598 | hCoV-19/Puerto Rico/PR-CDC-S185/2021 | North America / Puerto Rico | 2021-03-05 |
| EPI_ISL_1620599 | hCoV-19/Puerto Rico/PR-CDC-S189/2021 | North America / Puerto Rico | 2021-03-06 |
| EPI_ISL_1620600 | hCoV-19/Puerto Rico/PR-CDC-S190/2021 | North America / Puerto Rico | 2021-03-06 |
| EPI_ISL_1620601 | hCoV-19/Puerto Rico/PR-CDC-S193/2021 | North America / Puerto Rico | 2021-03-06 |
| EPI_ISL_1620602 | hCoV-19/Puerto Rico/PR-CDC-S194/2021 | North America / Puerto Rico | 2021-03-07 |
| EPI_ISL_1620603 | hCoV-19/Puerto Rico/PR-CDC-S199/2021 | North America / Puerto Rico | 2021-03-09 |
| EPI_ISL_1620604 | hCoV-19/Puerto Rico/PR-CDC-S207/2021 | North America / Puerto Rico | 2021-03-10 |
| EPI_ISL_1620605 | hCoV-19/Puerto Rico/PR-CDC-S176/2021 | North America / Puerto Rico | 2021-03-03 |
| EPI_ISL_1620606 | hCoV-19/Puerto Rico/PR-CDC-S187/2021 | North America / Puerto Rico | 2021-03-05 |
| EPI_ISL_1620607 | hCoV-19/Puerto Rico/PR-CDC-S218/2021 | North America / Puerto Rico | 2021-03-30 |
| EPI_ISL_1620608 | hCoV-19/Puerto Rico/PR-CDC-S245/2021 | North America / Puerto Rico | 2021-03-22 |
| EPI_ISL_1620609 | hCoV-19/Puerto Rico/PR-CDC-S177/2021 | North America / Puerto Rico | 2021-03-02 |
| EPI_ISL_1620610 | hCoV-19/Puerto Rico/PR-CDC-S178/2021 | North America / Puerto Rico | 2021-03-04 |
| EPI_ISL_1620611 | hCoV-19/Puerto Rico/PR-CDC-S181/2021 | North America / Puerto Rico | 2021-03-04 |
| EPI_ISL_1620612 | hCoV-19/Puerto Rico/PR-CDC-S182/2021 | North America / Puerto Rico | 2021-03-04 |
| EPI_ISL_1620613 | hCoV-19/Puerto Rico/PR-CDC-S184/2021 | North America / Puerto Rico | 2021-03-04 |
| EPI_ISL_1620614 | hCoV-19/Puerto Rico/PR-CDC-S188/2021 | North America / Puerto Rico | 2021-03-05 |
| EPI_ISL_1620615 | hCoV-19/Puerto Rico/PR-CDC-S191/2021 | North America / Puerto Rico | 2021-03-05 |
| EPI_ISL_1620616 | hCoV-19/Puerto Rico/PR-CDC-S192/2021 | North America / Puerto Rico | 2021-03-05 |
| EPI_ISL_1620617 | hCoV-19/Puerto Rico/PR-CDC-S196/2021 | North America / Puerto Rico | 2021-03-07 |
| EPI_ISL_1620618 | hCoV-19/Puerto Rico/PR-CDC-S197/2021 | North America / Puerto Rico | 2021-03-07 |
| EPI_ISL_1620619 | hCoV-19/Puerto Rico/PR-CDC-S201/2021 | North America / Puerto Rico | 2021-03-08 |
| EPI_ISL_1620620 | hCoV-19/Puerto Rico/PR-CDC-S202/2021 | North America / Puerto Rico | 2021-03-08 |
| EPI_ISL_1620621 | hCoV-19/Puerto Rico/PR-CDC-S204/2021 | North America / Puerto Rico | 2021-03-09 |
| EPI_ISL_1620622 | hCoV-19/Puerto Rico/PR-CDC-S205/2021 | North America / Puerto Rico | 2021-03-09 |
| EPI_ISL_1620623 | hCoV-19/Puerto Rico/PR-CDC-S209/2021 | North America / Puerto Rico | 2021-03-10 |
| EPI_ISL_1620624 | hCoV-19/Puerto Rico/PR-CDC-S211/2021 | North America / Puerto Rico | 2021-03-11 |
| EPI_ISL_1620625 | hCoV-19/Puerto Rico/PR-CDC-S215/2021 | North America / Puerto Rico | 2021-03-10 |
| EPI_ISL_1620626 | hCoV-19/Puerto Rico/PR-CDC-S216/2021 | North America / Puerto Rico | 2021-03-12 |
| EPI_ISL_1620627 | hCoV-19/Puerto Rico/PR-CDC-S206/2021 | North America / Puerto Rico | 2021-03-09 |
| EPI_ISL_1760210 | hCoV-19/Puerto Rico/PR-CDC-S268/2021 | North America / Puerto Rico | 2021-01-05 |
| EPI_ISL_1760211 | hCoV-19/Puerto Rico/PR-CDC-S269/2021 | North America / Puerto Rico | 2021-01-05 |
| EPI_ISL_1760212 | hCoV-19/Puerto Rico/PR-CDC-S270/2020 | North America / Puerto Rico | 2020-12-28 |
| EPI_ISL_1760213 | hCoV-19/Puerto Rico/PR-CDC-S271/2020 | North America / Puerto Rico | 2020-12-22 |
| EPI_ISL_1760214 | hCoV-19/Puerto Rico/PR-CDC-S272/2020 | North America / Puerto Rico | 2020-12-22 |
| EPI_ISL_1760215 | hCoV-19/Puerto Rico/PR-CDC-S273/2020 | North America / Puerto Rico | 2020-12-22 |
| EPI_ISL_1760216 | hCoV-19/Puerto Rico/PR-CDC-S274/2020 | North America / Puerto Rico | 2020-12-14 |
| EPI_ISL_1760217 | hCoV-19/Puerto Rico/PR-CDC-S275/2020 | North America / Puerto Rico | 2020-12-10 |
| EPI_ISL_1760218 | hCoV-19/Puerto Rico/PR-CDC-S276/2020 | North America / Puerto Rico | 2020-12-11 |
| EPI_ISL_1760219 | hCoV-19/Puerto Rico/PR-CDC-S277/2020 | North America / Puerto Rico | 2020-12-10 |
| EPI_ISL_1760220 | hCoV-19/Puerto Rico/PR-CDC-S278/2020 | North America / Puerto Rico | 2020-12-10 |
| EPI_ISL_1760221 | hCoV-19/Puerto Rico/PR-CDC-S279/2020 | North America / Puerto Rico | 2020-12-11 |
| EPI_ISL_1760222 | hCoV-19/Puerto Rico/PR-CDC-S280/2020 | North America / Puerto Rico | 2020-12-07 |
| EPI_ISL_1760223 | hCoV-19/Puerto Rico/PR-CDC-S281/2020 | North America / Puerto Rico | 2020-12-18 |
| EPI_ISL_1760224 | hCoV-19/Puerto Rico/PR-CDC-S282/2020 | North America / Puerto Rico | 2020-12-16 |
| EPI_ISL_1760225 | hCoV-19/Puerto Rico/PR-CDC-S283/2021 | North America / Puerto Rico | 2021-01-07 |
| EPI_ISL_1760226 | hCoV-19/Puerto Rico/PR-CDC-S284/2020 | North America / Puerto Rico | 2020-12-03 |
| EPI_ISL_1760227 | hCoV-19/Puerto Rico/PR-CDC-S285/2021 | North America / Puerto Rico | 2021-01-06 |
| EPI_ISL_1760228 | hCoV-19/Puerto Rico/PR-CDC-S286/2021 | North America / Puerto Rico | 2021-01-09 |
| EPI_ISL_1760229 | hCoV-19/Puerto Rico/PR-CDC-S287/2020 | North America / Puerto Rico | 2020-12-03 |
| EPI_ISL_1760230 | hCoV-19/Puerto Rico/PR-CDC-S288/2020 | North America / Puerto Rico | 2020-12-03 |
| EPI_ISL_1760231 | hCoV-19/Puerto Rico/PR-CDC-S289/2020 | North America / Puerto Rico | 2020-12-01 |
| EPI_ISL_1760232 | hCoV-19/Puerto Rico/PR-CDC-S290/2020 | North America / Puerto Rico | 2020-12-05 |
| EPI_ISL_1760233 | hCoV-19/Puerto Rico/PR-CDC-S291/2020 | North America / Puerto Rico | 2020-11-30 |
| EPI_ISL_1760234 | hCoV-19/Puerto Rico/PR-CDC-S292/2020 | North America / Puerto Rico | 2020-12-01 |
| EPI_ISL_1760235 | hCoV-19/Puerto Rico/PR-CDC-S293/2020 | North America / Puerto Rico | 2020-12-01 |
| EPI_ISL_1760236 | hCoV-19/Puerto Rico/PR-CDC-S294/2021 | North America / Puerto Rico | 2021-01-19 |
| EPI_ISL_1760237 | hCoV-19/Puerto Rico/PR-CDC-S295/2021 | North America / Puerto Rico | 2021-01-21 |
| EPI_ISL_1760238 | hCoV-19/Puerto Rico/PR-CDC-S296/2021 | North America / Puerto Rico | 2021-01-21 |
| EPI_ISL_1760239 | hCoV-19/Puerto Rico/PR-CDC-S297/2021 | North America / Puerto Rico | 2021-01-17 |
| EPI_ISL_1760240 | hCoV-19/Puerto Rico/PR-CDC-S298/2021 | North America / Puerto Rico | 2021-01-19 |
| EPI_ISL_1760241 | hCoV-19/Puerto Rico/PR-CDC-S299/2021 | North America / Puerto Rico | 2021-01-13 |
| EPI_ISL_1760242 | hCoV-19/Puerto Rico/PR-CDC-S300/2021 | North America / Puerto Rico | 2021-01-15 |
| EPI_ISL_1760243 | hCoV-19/Puerto Rico/PR-CDC-S301/2021 | North America / Puerto Rico | 2021-01-29 |
| EPI_ISL_1760244 | hCoV-19/Puerto Rico/PR-CDC-S302/2021 | North America / Puerto Rico | 2021-01-22 |
| EPI_ISL_1760245 | hCoV-19/Puerto Rico/PR-CDC-S303/2021 | North America / Puerto Rico | 2021-01-25 |
| EPI_ISL_1760246 | hCoV-19/Puerto Rico/PR-CDC-S304/2021 | North America / Puerto Rico | 2021-02-06 |
| EPI_ISL_1760247 | hCoV-19/Puerto Rico/PR-CDC-S305/2021 | North America / Puerto Rico | 2021-02-06 |
| EPI_ISL_1760248 | hCoV-19/Puerto Rico/PR-CDC-S306/2021 | North America / Puerto Rico | 2021-02-03 |
| EPI_ISL_1760249 | hCoV-19/Puerto Rico/PR-CDC-S307/2021 | North America / Puerto Rico | 2021-02-05 |
| EPI_ISL_1760250 | hCoV-19/Puerto Rico/PR-CDC-S308/2021 | North America / Puerto Rico | 2021-02-23 |
| EPI_ISL_1760251 | hCoV-19/Puerto Rico/PR-CDC-S309/2021 | North America / Puerto Rico | 2021-03-23 |
| EPI_ISL_1760252 | hCoV-19/Puerto Rico/PR-CDC-S310/2021 | North America / Puerto Rico | 2021-02-27 |
| EPI_ISL_1760253 | hCoV-19/Puerto Rico/PR-CDC-S311/2021 | North America / Puerto Rico | 2021-03-21 |
| EPI_ISL_1760254 | hCoV-19/Puerto Rico/PR-CDC-S312/2021 | North America / Puerto Rico | 2021-03-19 |
| EPI_ISL_1760255 | hCoV-19/Puerto Rico/PR-CDC-S313/2021 | North America / Puerto Rico | 2021-03-19 |
| EPI_ISL_1760256 | hCoV-19/Puerto Rico/PR-CDC-S314/2021 | North America / Puerto Rico | 2021-03-05 |
| EPI_ISL_1760257 | hCoV-19/Puerto Rico/PR-CDC-S315/2021 | North America / Puerto Rico | 2021-02-10 |
| EPI_ISL_1760258 | hCoV-19/Puerto Rico/PR-CDC-S316/2021 | North America / Puerto Rico | 2021-02-16 |
| EPI_ISL_1760259 | hCoV-19/Puerto Rico/PR-CDC-S317/2021 | North America / Puerto Rico | 2021-02-26 |
| EPI_ISL_1760260 | hCoV-19/Puerto Rico/PR-CDC-S318/2021 | North America / Puerto Rico | 2021-03-01 |
| EPI_ISL_1760261 | hCoV-19/Puerto Rico/PR-CDC-S319/2021 | North America / Puerto Rico | 2021-03-24 |
| EPI_ISL_1760262 | hCoV-19/Puerto Rico/PR-CDC-S320/2021 | North America / Puerto Rico | 2021-03-25 |
| EPI_ISL_1760263 | hCoV-19/Puerto Rico/PR-CDC-S321/2021 | North America / Puerto Rico | 2021-03-25 |
| EPI_ISL_1760264 | hCoV-19/Puerto Rico/PR-CDC-S322/2021 | North America / Puerto Rico | 2021-03-25 |
| EPI_ISL_1760265 | hCoV-19/Puerto Rico/PR-CDC-S323/2021 | North America / Puerto Rico | 2021-03-25 |
| EPI_ISL_1760266 | hCoV-19/Puerto Rico/PR-CDC-S324/2021 | North America / Puerto Rico | 2021-03-25 |
| EPI_ISL_1760267 | hCoV-19/Puerto Rico/PR-CDC-S325/2021 | North America / Puerto Rico | 2021-03-25 |
| EPI_ISL_1760268 | hCoV-19/Puerto Rico/PR-CDC-S326/2021 | North America / Puerto Rico | 2021-03-28 |
| EPI_ISL_1760269 | hCoV-19/Puerto Rico/PR-CDC-S327/2021 | North America / Puerto Rico | 2021-03-26 |
| EPI_ISL_1760270 | hCoV-19/Puerto Rico/PR-CDC-S328/2021 | North America / Puerto Rico | 2021-03-28 |
| EPI_ISL_1760271 | hCoV-19/Puerto Rico/PR-CDC-S329/2021 | North America / Puerto Rico | 2021-03-28 |
| EPI_ISL_1760272 | hCoV-19/Puerto Rico/PR-CDC-S330/2021 | North America / Puerto Rico | 2021-03-29 |
| EPI_ISL_1760273 | hCoV-19/Puerto Rico/PR-CDC-S331/2021 | North America / Puerto Rico | 2021-03-29 |
| EPI_ISL_1760274 | hCoV-19/Puerto Rico/PR-CDC-S332/2021 | North America / Puerto Rico | 2021-03-29 |
| EPI_ISL_1760275 | hCoV-19/Puerto Rico/PR-CDC-S334/2021 | North America / Puerto Rico | 2021-03-29 |
| EPI_ISL_1760276 | hCoV-19/Puerto Rico/PR-CDC-S335/2021 | North America / Puerto Rico | 2021-04-02 |
| EPI_ISL_1760277 | hCoV-19/Puerto Rico/PR-CDC-S336/2021 | North America / Puerto Rico | 2021-03-31 |
| EPI_ISL_1760278 | hCoV-19/Puerto Rico/PR-CDC-S337/2021 | North America / Puerto Rico | 2021-03-31 |
| EPI_ISL_1760279 | hCoV-19/Puerto Rico/PR-CDC-S338/2021 | North America / Puerto Rico | 2021-04-01 |
| EPI_ISL_1760280 | hCoV-19/Puerto Rico/PR-CDC-S339/2021 | North America / Puerto Rico | 2021-04-01 |
| EPI_ISL_1760281 | hCoV-19/Puerto Rico/PR-CDC-S340/2021 | North America / Puerto Rico | 2021-04-01 |
| EPI_ISL_1760282 | hCoV-19/Puerto Rico/PR-CDC-S341/2021 | North America / Puerto Rico | 2021-03-31 |
| EPI_ISL_1760283 | hCoV-19/Puerto Rico/PR-CDC-S342/2021 | North America / Puerto Rico | 2021-03-31 |
| EPI_ISL_1760284 | hCoV-19/Puerto Rico/PR-CDC-S343/2021 | North America / Puerto Rico | 2021-04-01 |
| EPI_ISL_1760285 | hCoV-19/Puerto Rico/PR-CDC-S344/2021 | North America / Puerto Rico | 2021-04-02 |
| EPI_ISL_1760286 | hCoV-19/Puerto Rico/PR-CDC-S345/2021 | North America / Puerto Rico | 2021-04-02 |
| EPI_ISL_1760287 | hCoV-19/Puerto Rico/PR-CDC-S346/2021 | North America / Puerto Rico | 2021-04-06 |
| EPI_ISL_1760288 | hCoV-19/Puerto Rico/PR-CDC-S347/2021 | North America / Puerto Rico | 2021-04-05 |
| EPI_ISL_1760289 | hCoV-19/Puerto Rico/PR-CDC-S348/2021 | North America / Puerto Rico | 2021-04-06 |
| EPI_ISL_1760290 | hCoV-19/Puerto Rico/PR-CDC-S349/2021 | North America / Puerto Rico | 2021-04-06 |
| EPI_ISL_1760291 | hCoV-19/Puerto Rico/PR-CDC-S350/2021 | North America / Puerto Rico | 2021-04-05 |
| EPI_ISL_1760292 | hCoV-19/Puerto Rico/PR-CDC-S351/2021 | North America / Puerto Rico | 2021-03-16 |
| EPI_ISL_1760293 | hCoV-19/Puerto Rico/PR-CDC-S352/2021 | North America / Puerto Rico | 2021-03-15 |
| EPI_ISL_1760294 | hCoV-19/Puerto Rico/PR-CDC-S353/2021 | North America / Puerto Rico | 2021-03-10 |
| EPI_ISL_1760295 | hCoV-19/Puerto Rico/PR-CDC-S354/2021 | North America / Puerto Rico | 2021-03-10 |
| EPI_ISL_1760296 | hCoV-19/Puerto Rico/PR-CDC-S355/2021 | North America / Puerto Rico | 2021-03-01 |
| EPI_ISL_1760297 | hCoV-19/Puerto Rico/PR-CDC-S356/2021 | North America / Puerto Rico | 2021-03-02 |
| EPI_ISL_1760298 | hCoV-19/Puerto Rico/PR-CDC-S357/2021 | North America / Puerto Rico | 2021-03-02 |
| EPI_ISL_1760299 | hCoV-19/Puerto Rico/PR-CDC-S358/2021 | North America / Puerto Rico | 2021-03-02 |
| EPI_ISL_2426853 | hCoV-19/Puerto Rico/PR-CDC-S377/2020 | North America / Puerto Rico | 2020-11-05 |
| EPI_ISL_2426854 | hCoV-19/Puerto Rico/PR-CDC-S414/2021 | North America / Puerto Rico | 2021-04-26 |
| EPI_ISL_2426855 | hCoV-19/Puerto Rico/PR-CDC-S407/2021 | North America / Puerto Rico | 2021-04-24 |
| EPI_ISL_2426856 | hCoV-19/Puerto Rico/PR-CDC-S409/2021 | North America / Puerto Rico | 2021-04-24 |
| EPI_ISL_2426857 | hCoV-19/Puerto Rico/PR-CDC-S415/2021 | North America / Puerto Rico | 2021-04-24 |
| EPI_ISL_2426858 | hCoV-19/Puerto Rico/PR-CDC-S433/2021 | North America / Puerto Rico | 2021-04-09 |
| EPI_ISL_2426859 | hCoV-19/Puerto Rico/PR-CDC-S438/2021 | North America / Puerto Rico | 2021-04-17 |
| EPI_ISL_2426860 | hCoV-19/Puerto Rico/PR-CDC-S410/2021 | North America / Puerto Rico | 2021-04-24 |
| EPI_ISL_2426861 | hCoV-19/Puerto Rico/PR-CDC-S441/2021 | North America / Puerto Rico | 2021-04-07 |
| EPI_ISL_2426862 | hCoV-19/Puerto Rico/PR-CDC-S443/2021 | North America / Puerto Rico | 2021-04-07 |
| EPI_ISL_2426863 | hCoV-19/Puerto Rico/PR-CDC-S445/2021 | North America / Puerto Rico | 2021-04-05 |
| EPI_ISL_2426864 | hCoV-19/Puerto Rico/PR-CDC-S448/2021 | North America / Puerto Rico | 2021-04-08 |
| EPI_ISL_2426865 | hCoV-19/Puerto Rico/PR-CDC-S449/2021 | North America / Puerto Rico | 2021-04-22 |
| EPI_ISL_2426866 | hCoV-19/Puerto Rico/PR-CDC-S404/2021 | North America / Puerto Rico | 2021-04-12 |
| EPI_ISL_2426867 | hCoV-19/Puerto Rico/PR-CDC-S405/2021 | North America / Puerto Rico | 2021-04-22 |
| EPI_ISL_2426868 | hCoV-19/Puerto Rico/PR-CDC-S411/2021 | North America / Puerto Rico | 2021-04-23 |
| EPI_ISL_2426869 | hCoV-19/Puerto Rico/PR-CDC-S413/2021 | North America / Puerto Rico | 2021-04-24 |
| EPI_ISL_2426870 | hCoV-19/Puerto Rico/PR-CDC-S416/2021 | North America / Puerto Rico | 2021-04-28 |
| EPI_ISL_2426871 | hCoV-19/Puerto Rico/PR-CDC-S417/2021 | North America / Puerto Rico | 2021-04-12 |
| EPI_ISL_2426872 | hCoV-19/Puerto Rico/PR-CDC-S421/2021 | North America / Puerto Rico | 2021-04-13 |
| EPI_ISL_2426873 | hCoV-19/Puerto Rico/PR-CDC-S423/2021 | North America / Puerto Rico | 2021-04-08 |
| EPI_ISL_2426874 | hCoV-19/Puerto Rico/PR-CDC-S430/2021 | North America / Puerto Rico | 2021-04-07 |
| EPI_ISL_2426875 | hCoV-19/Puerto Rico/PR-CDC-S431/2021 | North America / Puerto Rico | 2021-04-11 |
| EPI_ISL_2426876 | hCoV-19/Puerto Rico/PR-CDC-S432/2021 | North America / Puerto Rico | 2021-04-10 |
| EPI_ISL_2426877 | hCoV-19/Puerto Rico/PR-CDC-S434/2021 | North America / Puerto Rico | 2021-04-10 |
| EPI_ISL_2426878 | hCoV-19/Puerto Rico/PR-CDC-S435/2021 | North America / Puerto Rico | 2021-04-17 |
| EPI_ISL_2426879 | hCoV-19/Puerto Rico/PR-CDC-S436/2021 | North America / Puerto Rico | 2021-04-16 |
| EPI_ISL_2426880 | hCoV-19/Puerto Rico/PR-CDC-S444/2021 | North America / Puerto Rico | 2021-04-05 |
| EPI_ISL_2426881 | hCoV-19/Puerto Rico/PR-CDC-S446/2021 | North America / Puerto Rico | 2021-04-05 |
| EPI_ISL_2426882 | hCoV-19/Puerto Rico/PR-CDC-S412/2021 | North America / Puerto Rico | 2021-04-23 |
| EPI_ISL_2426883 | hCoV-19/Puerto Rico/PR-CDC-S408/2021 | North America / Puerto Rico | 2021-04-23 |
| EPI_ISL_2426884 | hCoV-19/Puerto Rico/PR-CDC-S427/2021 | North America / Puerto Rico | 2021-04-16 |
| EPI_ISL_2426885 | hCoV-19/Puerto Rico/PR-CDC-S420/2021 | North America / Puerto Rico | 2021-04-12 |
| EPI_ISL_2426886 | hCoV-19/Puerto Rico/PR-CDC-S406/2021 | North America / Puerto Rico | 2021-04-21 |
| EPI_ISL_2426887 | hCoV-19/Puerto Rico/PR-CDC-S424/2021 | North America / Puerto Rico | 2021-04-07 |
| EPI_ISL_2426888 | hCoV-19/Puerto Rico/PR-CDC-S425/2021 | North America / Puerto Rico | 2021-04-07 |
| EPI_ISL_2426889 | hCoV-19/Puerto Rico/PR-CDC-S426/2021 | North America / Puerto Rico | 2021-04-07 |
| EPI_ISL_2426890 | hCoV-19/Puerto Rico/PR-CDC-S429/2021 | North America / Puerto Rico | 2021-04-09 |
| EPI_ISL_2426891 | hCoV-19/Puerto Rico/PR-CDC-S437/2021 | North America / Puerto Rico | 2021-04-17 |
| EPI_ISL_2426892 | hCoV-19/Puerto Rico/PR-CDC-S442/2021 | North America / Puerto Rico | 2021-04-07 |
| EPI_ISL_2426894 | hCoV-19/Puerto Rico/PR-CDC-S428/2021 | North America / Puerto Rico | 2021-04-14 |
| EPI_ISL_2426895 | hCoV-19/Puerto Rico/PR-CDC-S439/2021 | North America / Puerto Rico | 2021-04-20 |
| EPI_ISL_2426896 | hCoV-19/Puerto Rico/PR-CDC-S440/2021 | North America / Puerto Rico | 2021-04-07 |
| EPI_ISL_2426897 | hCoV-19/Puerto Rico/PR-CDC-S447/2021 | North America / Puerto Rico | 2021-04-08 |
| EPI_ISL_2426898 | hCoV-19/Puerto Rico/PR-CDC-S359/2020 | North America / Puerto Rico | 2020-10-26 |
| EPI_ISL_2426899 | hCoV-19/Puerto Rico/PR-CDC-S360/2020 | North America / Puerto Rico | 2020-10-27 |
| EPI_ISL_2426900 | hCoV-19/Puerto Rico/PR-CDC-S361/2020 | North America / Puerto Rico | 2020-10-21 |
| EPI_ISL_2426901 | hCoV-19/Puerto Rico/PR-CDC-S362/2020 | North America / Puerto Rico | 2020-10-21 |
| EPI_ISL_2426902 | hCoV-19/Puerto Rico/PR-CDC-S363/2020 | North America / Puerto Rico | 2020-10-27 |
| EPI_ISL_2426903 | hCoV-19/Puerto Rico/PR-CDC-S364/2020 | North America / Puerto Rico | 2020-11-02 |
| EPI_ISL_2426904 | hCoV-19/Puerto Rico/PR-CDC-S365/2020 | North America / Puerto Rico | 2020-11-03 |
| EPI_ISL_2426905 | hCoV-19/Puerto Rico/PR-CDC-S366/2020 | North America / Puerto Rico | 2020-10-30 |
| EPI_ISL_2426906 | hCoV-19/Puerto Rico/PR-CDC-S367/2020 | North America / Puerto Rico | 2020-10-30 |
| EPI_ISL_2426907 | hCoV-19/Puerto Rico/PR-CDC-S368/2020 | North America / Puerto Rico | 2020-10-30 |
| EPI_ISL_2426908 | hCoV-19/Puerto Rico/PR-CDC-S369/2020 | North America / Puerto Rico | 2020-10-31 |
| EPI_ISL_2426909 | hCoV-19/Puerto Rico/PR-CDC-S370/2020 | North America / Puerto Rico | 2020-10-30 |
| EPI_ISL_2426910 | hCoV-19/Puerto Rico/PR-CDC-S371/2020 | North America / Puerto Rico | 2020-11-01 |
| EPI_ISL_2426911 | hCoV-19/Puerto Rico/PR-CDC-S372/2020 | North America / Puerto Rico | 2020-10-30 |
| EPI_ISL_2426912 | hCoV-19/Puerto Rico/PR-CDC-S373/2020 | North America / Puerto Rico | 2020-10-31 |
| EPI_ISL_2426913 | hCoV-19/Puerto Rico/PR-CDC-S374/2020 | North America / Puerto Rico | 2020-11-08 |
| EPI_ISL_2426914 | hCoV-19/Puerto Rico/PR-CDC-S375/2020 | North America / Puerto Rico | 2020-11-05 |
| EPI_ISL_2426915 | hCoV-19/Puerto Rico/PR-CDC-S376/2020 | North America / Puerto Rico | 2020-11-04 |
| EPI_ISL_2426916 | hCoV-19/Puerto Rico/PR-CDC-S378/2020 | North America / Puerto Rico | 2020-11-04 |
| EPI_ISL_2426917 | hCoV-19/Puerto Rico/PR-CDC-S379/2020 | North America / Puerto Rico | 2020-11-04 |
| EPI_ISL_2426918 | hCoV-19/Puerto Rico/PR-CDC-S380/2020 | North America / Puerto Rico | 2020-11-05 |
| EPI_ISL_2426919 | hCoV-19/Puerto Rico/PR-CDC-S381/2020 | North America / Puerto Rico | 2020-11-08 |
| EPI_ISL_2426920 | hCoV-19/Puerto Rico/PR-CDC-S382/2020 | North America / Puerto Rico | 2020-11-11 |
| EPI_ISL_2426921 | hCoV-19/Puerto Rico/PR-CDC-S383/2020 | North America / Puerto Rico | 2020-11-09 |
| EPI_ISL_2426922 | hCoV-19/Puerto Rico/PR-CDC-S384/2020 | North America / Puerto Rico | 2020-11-11 |
| EPI_ISL_2426923 | hCoV-19/Puerto Rico/PR-CDC-S385/2020 | North America / Puerto Rico | 2020-11-16 |
| EPI_ISL_2426924 | hCoV-19/Puerto Rico/PR-CDC-S386/2020 | North America / Puerto Rico | 2020-11-16 |
| EPI_ISL_2426925 | hCoV-19/Puerto Rico/PR-CDC-S387/2020 | North America / Puerto Rico | 2020-11-17 |
| EPI_ISL_2426926 | hCoV-19/Puerto Rico/PR-CDC-S388/2020 | North America / Puerto Rico | 2020-11-16 |
| EPI_ISL_2426927 | hCoV-19/Puerto Rico/PR-CDC-S389/2020 | North America / Puerto Rico | 2020-11-14 |
| EPI_ISL_2426928 | hCoV-19/Puerto Rico/PR-CDC-S390/2020 | North America / Puerto Rico | 2020-11-13 |
| EPI_ISL_2426929 | hCoV-19/Puerto Rico/PR-CDC-S391/2020 | North America / Puerto Rico | 2020-11-14 |
| EPI_ISL_2426930 | hCoV-19/Puerto Rico/PR-CDC-S392/2020 | North America / Puerto Rico | 2020-11-10 |
| EPI_ISL_2426931 | hCoV-19/Puerto Rico/PR-CDC-S393/2020 | North America / Puerto Rico | 2020-11-15 |
| EPI_ISL_2426932 | hCoV-19/Puerto Rico/PR-CDC-S394/2020 | North America / Puerto Rico | 2020-11-13 |
| EPI_ISL_2426933 | hCoV-19/Puerto Rico/PR-CDC-S395/2020 | North America / Puerto Rico | 2020-11-09 |
| EPI_ISL_2426934 | hCoV-19/Puerto Rico/PR-CDC-S396/2020 | North America / Puerto Rico | 2020-11-23 |
| EPI_ISL_2426935 | hCoV-19/Puerto Rico/PR-CDC-S397/2020 | North America / Puerto Rico | 2020-11-20 |
| EPI_ISL_2426936 | hCoV-19/Puerto Rico/PR-CDC-S398/2020 | North America / Puerto Rico | 2020-11-23 |
| EPI_ISL_2426937 | hCoV-19/Puerto Rico/PR-CDC-S399/2020 | North America / Puerto Rico | 2020-11-20 |
| EPI_ISL_2426938 | hCoV-19/Puerto Rico/PR-CDC-S400/2020 | North America / Puerto Rico | 2020-11-21 |
| EPI_ISL_2426939 | hCoV-19/Puerto Rico/PR-CDC-S401/2020 | North America / Puerto Rico | 2020-12-01 |
| EPI_ISL_2426940 | hCoV-19/Puerto Rico/PR-CDC-S402/2020 | North America / Puerto Rico | 2020-12-01 |
| EPI_ISL_2426941 | hCoV-19/Puerto Rico/PR-CDC-S403/2021 | North America / Puerto Rico | 2021-01-26 |
| EPI_ISL_2426942 | hCoV-19/Puerto Rico/PR-CDC-S418/2020 | North America / Puerto Rico | 2020-12-16 |
| EPI_ISL_2426943 | hCoV-19/Puerto Rico/PR-CDC-S419/2020 | North America / Puerto Rico | 2020-12-05 |
| EPI_ISL_2688259 | hCoV-19/Puerto Rico/PR-CDC-S451/2021 | North America / Puerto Rico | 2021-05-12 |
| EPI_ISL_2688260 | hCoV-19/Puerto Rico/PR-CDC-S452/2021 | North America / Puerto Rico | 2021-05-17 |
| EPI_ISL_2688261 | hCoV-19/Puerto Rico/PR-CDC-S453/2021 | North America / Puerto Rico | 2021-04-20 |
| EPI_ISL_2688262 | hCoV-19/Puerto Rico/PR-CDC-S454/2021 | North America / Puerto Rico | 2021-04-20 |
| EPI_ISL_2688263 | hCoV-19/Puerto Rico/PR-CDC-S455/2021 | North America / Puerto Rico | 2021-04-19 |
| EPI_ISL_2688264 | hCoV-19/Puerto Rico/PR-CDC-S456/2021 | North America / Puerto Rico | 2021-04-19 |
| EPI_ISL_2688265 | hCoV-19/Puerto Rico/PR-CDC-S457/2021 | North America / Puerto Rico | 2021-04-19 |
| EPI_ISL_2688266 | hCoV-19/Puerto Rico/PR-CDC-S458/2021 | North America / Puerto Rico | 2021-04-28 |
| EPI_ISL_2688267 | hCoV-19/Puerto Rico/PR-CDC-S459/2020 | North America / Puerto Rico | 2020-11-25 |
| EPI_ISL_2688268 | hCoV-19/Puerto Rico/PR-CDC-S460/2021 | North America / Puerto Rico | 2021-04-13 |
| EPI_ISL_2688269 | hCoV-19/Puerto Rico/PR-CDC-S461/2021 | North America / Puerto Rico | 2021-04-10 |
| EPI_ISL_2688270 | hCoV-19/Puerto Rico/PR-CDC-S462/2021 | North America / Puerto Rico | 2021-04-16 |
| EPI_ISL_2688271 | hCoV-19/Puerto Rico/PR-CDC-S463/2021 | North America / Puerto Rico | 2021-05-07 |
| EPI_ISL_2688272 | hCoV-19/Puerto Rico/PR-CDC-S464/2021 | North America / Puerto Rico | 2021-04-29 |
| EPI_ISL_2688273 | hCoV-19/Puerto Rico/PR-CDC-S465/2021 | North America / Puerto Rico | 2021-05-04 |
| EPI_ISL_2688274 | hCoV-19/Puerto Rico/PR-CDC-S466/2021 | North America / Puerto Rico | 2021-05-11 |
| EPI_ISL_2688275 | hCoV-19/Puerto Rico/PR-CDC-S467/2021 | North America / Puerto Rico | 2021-05-05 |
| EPI_ISL_2688276 | hCoV-19/Puerto Rico/PR-CDC-S468/2021 | North America / Puerto Rico | 2021-05-05 |
| EPI_ISL_2688277 | hCoV-19/Puerto Rico/PR-CDC-S469/2020 | North America / Puerto Rico | 2020-10-22 |
| EPI_ISL_2688278 | hCoV-19/Puerto Rico/PR-CDC-S470/2021 | North America / Puerto Rico | 2021-04-09 |
| EPI_ISL_2688279 | hCoV-19/Puerto Rico/PR-CDC-S471/2021 | North America / Puerto Rico | 2021-04-06 |
| EPI_ISL_2688280 | hCoV-19/Puerto Rico/PR-CDC-S472/2021 | North America / Puerto Rico | 2021-04-08 |
| EPI_ISL_2688281 | hCoV-19/Puerto Rico/PR-CDC-S473/2021 | North America / Puerto Rico | 2021-04-08 |
| EPI_ISL_2688282 | hCoV-19/Puerto Rico/PR-CDC-S474/2021 | North America / Puerto Rico | 2021-04-08 |
| EPI_ISL_2688283 | hCoV-19/Puerto Rico/PR-CDC-S475/2021 | North America / Puerto Rico | 2021-04-07 |
| EPI_ISL_2688284 | hCoV-19/Puerto Rico/PR-CDC-S476/2021 | North America / Puerto Rico | 2021-04-21 |
| EPI_ISL_2688285 | hCoV-19/Puerto Rico/PR-CDC-S477/2021 | North America / Puerto Rico | 2021-04-22 |
| EPI_ISL_2688286 | hCoV-19/Puerto Rico/PR-CDC-S478/2021 | North America / Puerto Rico | 2021-04-15 |
| EPI_ISL_2688287 | hCoV-19/Puerto Rico/PR-CDC-S479/2021 | North America / Puerto Rico | 2021-04-14 |
| EPI_ISL_2688288 | hCoV-19/Puerto Rico/PR-CDC-S480/2021 | North America / Puerto Rico | 2021-04-09 |
| EPI_ISL_2688289 | hCoV-19/Puerto Rico/PR-CDC-S481/2021 | North America / Puerto Rico | 2021-05-01 |
| EPI_ISL_2688290 | hCoV-19/Puerto Rico/PR-CDC-S482/2021 | North America / Puerto Rico | 2021-05-02 |
| EPI_ISL_2688291 | hCoV-19/Puerto Rico/PR-CDC-S483/2021 | North America / Puerto Rico | 2021-05-03 |
| EPI_ISL_2688292 | hCoV-19/Puerto Rico/PR-CDC-S484/2021 | North America / Puerto Rico | 2021-05-02 |
| EPI_ISL_2688293 | hCoV-19/Puerto Rico/PR-CDC-S485/2021 | North America / Puerto Rico | 2021-05-02 |
| EPI_ISL_2688294 | hCoV-19/Puerto Rico/PR-CDC-S486/2021 | North America / Puerto Rico | 2021-04-29 |
| EPI_ISL_2688295 | hCoV-19/Puerto Rico/PR-CDC-S487/2021 | North America / Puerto Rico | 2021-05-03 |
| EPI_ISL_2688296 | hCoV-19/Puerto Rico/PR-CDC-S488/2021 | North America / Puerto Rico | 2021-05-02 |
| EPI_ISL_2688297 | hCoV-19/Puerto Rico/PR-CDC-S489/2021 | North America / Puerto Rico | 2021-04-17 |
| EPI_ISL_2688298 | hCoV-19/Puerto Rico/PR-CDC-S490/2021 | North America / Puerto Rico | 2021-05-03 |
| EPI_ISL_2688299 | hCoV-19/Puerto Rico/PR-CDC-S491/2021 | North America / Puerto Rico | 2021-04-29 |
| EPI_ISL_2688300 | hCoV-19/Puerto Rico/PR-CDC-S492/2021 | North America / Puerto Rico | 2021-05-03 |
| EPI_ISL_2688301 | hCoV-19/Puerto Rico/PR-CDC-S493/2021 | North America / Puerto Rico | 2021-05-01 |
| EPI_ISL_2688302 | hCoV-19/Puerto Rico/PR-CDC-S494/2021 | North America / Puerto Rico | 2021-05-01 |
| EPI_ISL_2688303 | hCoV-19/Puerto Rico/PR-CDC-S495/2021 | North America / Puerto Rico | 2021-05-03 |
| EPI_ISL_2688304 | hCoV-19/Puerto Rico/PR-CDC-S496/2021 | North America / Puerto Rico | 2021-05-17 |
| EPI_ISL_2688305 | hCoV-19/Puerto Rico/PR-CDC-S497/2021 | North America / Puerto Rico | 2021-05-18 |
| EPI_ISL_2688306 | hCoV-19/Puerto Rico/PR-CDC-S498/2021 | North America / Puerto Rico | 2021-05-18 |
| EPI_ISL_2688307 | hCoV-19/Puerto Rico/PR-CDC-S499/2021 | North America / Puerto Rico | 2021-05-19 |
| EPI_ISL_2688308 | hCoV-19/Puerto Rico/PR-CDC-S500/2021 | North America / Puerto Rico | 2021-05-19 |
| EPI_ISL_2688309 | hCoV-19/Puerto Rico/PR-CDC-S501/2021 | North America / Puerto Rico | 2021-05-19 |
| EPI_ISL_2688310 | hCoV-19/Puerto Rico/PR-CDC-S502/2021 | North America / Puerto Rico | 2021-05-19 |
| EPI_ISL_2688311 | hCoV-19/Puerto Rico/PR-CDC-S503/2021 | North America / Puerto Rico | 2021-05-20 |
| EPI_ISL_2688312 | hCoV-19/Puerto Rico/PR-CDC-S504/2021 | North America / Puerto Rico | 2021-05-20 |
| EPI_ISL_2688313 | hCoV-19/Puerto Rico/PR-CDC-S505/2021 | North America / Puerto Rico | 2021-05-21 |
| EPI_ISL_2688314 | hCoV-19/Puerto Rico/PR-CDC-S506/2021 | North America / Puerto Rico | 2021-05-21 |
| EPI_ISL_2688315 | hCoV-19/Puerto Rico/PR-CDC-S507/2021 | North America / Puerto Rico | 2021-05-21 |
| EPI_ISL_2688316 | hCoV-19/Puerto Rico/PR-CDC-S508/2021 | North America / Puerto Rico | 2021-05-21 |
| EPI_ISL_2688317 | hCoV-19/Puerto Rico/PR-CDC-S509/2021 | North America / Puerto Rico | 2021-05-22 |
| EPI_ISL_2688318 | hCoV-19/Puerto Rico/PR-CDC-S510/2021 | North America / Puerto Rico | 2021-05-22 |
| EPI_ISL_2688319 | hCoV-19/Puerto Rico/PR-CDC-S511/2021 | North America / Puerto Rico | 2021-05-24 |
| EPI_ISL_2756557 | hCoV-19/Puerto Rico/PR-CDC-S450/2021 | North America / Puerto Rico | 2021-06-07 |
| EPI_ISL_3132189 | hCoV-19/Puerto Rico/PR-CDC-S512/2021 | North America / Puerto Rico | 2021-06-03 |
| EPI_ISL_3132190 | hCoV-19/Puerto Rico/PR-CDC-S513/2021 | North America / Puerto Rico | 2021-06-16 |
| EPI_ISL_3132191 | hCoV-19/Puerto Rico/PR-CDC-S518/2021 | North America / Puerto Rico | 2021-06-27 |
| EPI_ISL_3132192 | hCoV-19/Puerto Rico/PR-CDC-S519/2021 | North America / Puerto Rico | 2021-06-28 |
| EPI_ISL_3132193 | hCoV-19/Puerto Rico/PR-CDC-S520/2021 | North America / Puerto Rico | 2021-06-28 |
| EPI_ISL_3132194 | hCoV-19/Puerto Rico/PR-CDC-S521/2021 | North America / Puerto Rico | 2021-06-29 |
| EPI_ISL_3132195 | hCoV-19/Puerto Rico/PR-CDC-S522/2021 | North America / Puerto Rico | 2021-06-29 |
| EPI_ISL_3132196 | hCoV-19/Puerto Rico/PR-CDC-S523/2021 | North America / Puerto Rico | 2021-07-12 |
| EPI_ISL_3155725 | hCoV-19/Puerto Rico/PR-CDC-S514/2021 | North America / Puerto Rico | 2021-06-20 |
| EPI_ISL_3155726 | hCoV-19/Puerto Rico/PR-CDC-S515/2021 | North America / Puerto Rico | 2021-06-18 |
| EPI_ISL_3155727 | hCoV-19/Puerto Rico/PR-CDC-S516/2021 | North America / Puerto Rico | 2021-06-19 |
| EPI_ISL_3155728 | hCoV-19/Puerto Rico/PR-CDC-S517/2021 | North America / Puerto Rico | 2021-06-13 |
| EPI_ISL_3718734 | hCoV-19/Puerto Rico/PR-CDC-S546/2021 | North America / Puerto Rico | 2021-06-15 |
| EPI_ISL_3718735 | hCoV-19/Puerto Rico/PR-CDC-S574/2021 | North America / Puerto Rico | 2021-07-05 |
| EPI_ISL_3718736 | hCoV-19/Puerto Rico/PR-CDC-S566/2021 | North America / Puerto Rico | 2021-07-20 |
| EPI_ISL_3718737 | hCoV-19/Puerto Rico/PR-CDC-S549/2021 | North America / Puerto Rico | 2021-07-21 |
| EPI_ISL_3718738 | hCoV-19/Puerto Rico/PR-CDC-S560/2021 | North America / Puerto Rico | 2021-07-21 |
| EPI_ISL_3718739 | hCoV-19/Puerto Rico/PR-CDC-S561/2021 | North America / Puerto Rico | 2021-07-21 |
| EPI_ISL_3718740 | hCoV-19/Puerto Rico/PR-CDC-S562/2021 | North America / Puerto Rico | 2021-07-21 |
| EPI_ISL_3718741 | hCoV-19/Puerto Rico/PR-CDC-S563/2021 | North America / Puerto Rico | 2021-07-21 |
| EPI_ISL_3718742 | hCoV-19/Puerto Rico/PR-CDC-S564/2021 | North America / Puerto Rico | 2021-07-21 |
| EPI_ISL_3718743 | hCoV-19/Puerto Rico/PR-CDC-S565/2021 | North America / Puerto Rico | 2021-07-21 |
| EPI_ISL_3718744 | hCoV-19/Puerto Rico/PR-CDC-S525/2021 | North America / Puerto Rico | 2021-07-22 |
| EPI_ISL_3718745 | hCoV-19/Puerto Rico/PR-CDC-S567/2021 | North America / Puerto Rico | 2021-07-22 |
| EPI_ISL_3718746 | hCoV-19/Puerto Rico/PR-CDC-S568/2021 | North America / Puerto Rico | 2021-07-22 |
| EPI_ISL_3718747 | hCoV-19/Puerto Rico/PR-CDC-S569/2021 | North America / Puerto Rico | 2021-07-22 |
| EPI_ISL_3718748 | hCoV-19/Puerto Rico/PR-CDC-S570/2021 | North America / Puerto Rico | 2021-07-22 |
| EPI_ISL_3718749 | hCoV-19/Puerto Rico/PR-CDC-S571/2021 | North America / Puerto Rico | 2021-07-22 |
| EPI_ISL_3718750 | hCoV-19/Puerto Rico/PR-CDC-S524/2021 | North America / Puerto Rico | 2021-07-23 |
| EPI_ISL_3718751 | hCoV-19/Puerto Rico/PR-CDC-S528/2021 | North America / Puerto Rico | 2021-07-23 |
| EPI_ISL_3718752 | hCoV-19/Puerto Rico/PR-CDC-S529/2021 | North America / Puerto Rico | 2021-07-25 |
| EPI_ISL_3718753 | hCoV-19/Puerto Rico/PR-CDC-S530/2021 | North America / Puerto Rico | 2021-07-25 |
| EPI_ISL_3718754 | hCoV-19/Puerto Rico/PR-CDC-S533/2021 | North America / Puerto Rico | 2021-07-25 |
| EPI_ISL_3718755 | hCoV-19/Puerto Rico/PR-CDC-S526/2021 | North America / Puerto Rico | 2021-07-26 |
| EPI_ISL_3718756 | hCoV-19/Puerto Rico/PR-CDC-S527/2021 | North America / Puerto Rico | 2021-07-27 |
| EPI_ISL_3718757 | hCoV-19/Puerto Rico/PR-CDC-S575/2021 | North America / Puerto Rico | 2021-07-27 |
| EPI_ISL_3718758 | hCoV-19/Puerto Rico/PR-CDC-S576/2021 | North America / Puerto Rico | 2021-07-27 |
| EPI_ISL_3718759 | hCoV-19/Puerto Rico/PR-CDC-S589/2021 | North America / Puerto Rico | 2021-07-27 |
| EPI_ISL_3718760 | hCoV-19/Puerto Rico/PR-CDC-S590/2021 | North America / Puerto Rico | 2021-07-27 |
| EPI_ISL_3718761 | hCoV-19/Puerto Rico/PR-CDC-S593/2021 | North America / Puerto Rico | 2021-07-27 |
| EPI_ISL_3718762 | hCoV-19/Puerto Rico/PR-CDC-S595/2021 | North America / Puerto Rico | 2021-07-27 |
| EPI_ISL_3718763 | hCoV-19/Puerto Rico/PR-CDC-S572/2021 | North America / Puerto Rico | 2021-07-28 |
| EPI_ISL_3718764 | hCoV-19/Puerto Rico/PR-CDC-S573/2021 | North America / Puerto Rico | 2021-07-28 |
| EPI_ISL_3718765 | hCoV-19/Puerto Rico/PR-CDC-S577/2021 | North America / Puerto Rico | 2021-07-28 |
| EPI_ISL_3718766 | hCoV-19/Puerto Rico/PR-CDC-S578/2021 | North America / Puerto Rico | 2021-07-28 |
| EPI_ISL_3718767 | hCoV-19/Puerto Rico/PR-CDC-S579/2021 | North America / Puerto Rico | 2021-07-28 |
| EPI_ISL_3718768 | hCoV-19/Puerto Rico/PR-CDC-S580/2021 | North America / Puerto Rico | 2021-07-28 |
| EPI_ISL_3718769 | hCoV-19/Puerto Rico/PR-CDC-S581/2021 | North America / Puerto Rico | 2021-07-28 |
| EPI_ISL_3718770 | hCoV-19/Puerto Rico/PR-CDC-S582/2021 | North America / Puerto Rico | 2021-07-28 |
| EPI_ISL_3718771 | hCoV-19/Puerto Rico/PR-CDC-S583/2021 | North America / Puerto Rico | 2021-07-28 |
| EPI_ISL_3718772 | hCoV-19/Puerto Rico/PR-CDC-S584/2021 | North America / Puerto Rico | 2021-07-28 |
| EPI_ISL_3718773 | hCoV-19/Puerto Rico/PR-CDC-S585/2021 | North America / Puerto Rico | 2021-07-28 |
| EPI_ISL_3718774 | hCoV-19/Puerto Rico/PR-CDC-S586/2021 | North America / Puerto Rico | 2021-07-28 |
| EPI_ISL_3718775 | hCoV-19/Puerto Rico/PR-CDC-S587/2021 | North America / Puerto Rico | 2021-07-28 |
| EPI_ISL_3718776 | hCoV-19/Puerto Rico/PR-CDC-S588/2021 | North America / Puerto Rico | 2021-07-28 |
| EPI_ISL_3718777 | hCoV-19/Puerto Rico/PR-CDC-S591/2021 | North America / Puerto Rico | 2021-07-28 |
| EPI_ISL_3718778 | hCoV-19/Puerto Rico/PR-CDC-S592/2021 | North America / Puerto Rico | 2021-07-28 |
| EPI_ISL_3718779 | hCoV-19/Puerto Rico/PR-CDC-S594/2021 | North America / Puerto Rico | 2021-07-28 |
| EPI_ISL_3718780 | hCoV-19/Puerto Rico/PR-CDC-S596/2021 | North America / Puerto Rico | 2021-07-28 |
| EPI_ISL_3718781 | hCoV-19/Puerto Rico/PR-CDC-S532/2021 | North America / Puerto Rico | 2021-07-29 |
| EPI_ISL_3718782 | hCoV-19/Puerto Rico/PR-CDC-S538/2021 | North America / Puerto Rico | 2021-07-29 |
| EPI_ISL_3718783 | hCoV-19/Puerto Rico/PR-CDC-S531/2021 | North America / Puerto Rico | 2021-07-30 |
| EPI_ISL_3718784 | hCoV-19/Puerto Rico/PR-CDC-S534/2021 | North America / Puerto Rico | 2021-07-30 |
| EPI_ISL_3718785 | hCoV-19/Puerto Rico/PR-CDC-S535/2021 | North America / Puerto Rico | 2021-07-30 |
| EPI_ISL_3718786 | hCoV-19/Puerto Rico/PR-CDC-S537/2021 | North America / Puerto Rico | 2021-07-30 |
| EPI_ISL_3718787 | hCoV-19/Puerto Rico/PR-CDC-S536/2021 | North America / Puerto Rico | 2021-07-31 |
| EPI_ISL_3718788 | hCoV-19/Puerto Rico/PR-CDC-S540/2021 | North America / Puerto Rico | 2021-08-04 |
| EPI_ISL_3718789 | hCoV-19/Puerto Rico/PR-CDC-S541/2021 | North America / Puerto Rico | 2021-08-04 |
| EPI_ISL_3718790 | hCoV-19/Puerto Rico/PR-CDC-S539/2021 | North America / Puerto Rico | 2021-08-05 |
| EPI_ISL_3718791 | hCoV-19/Puerto Rico/PR-CDC-S542/2021 | North America / Puerto Rico | 2021-08-05 |
| EPI_ISL_3718792 | hCoV-19/Puerto Rico/PR-CDC-S543/2021 | North America / Puerto Rico | 2021-08-06 |
| EPI_ISL_3718793 | hCoV-19/Puerto Rico/PR-CDC-S544/2021 | North America / Puerto Rico | 2021-08-09 |
| EPI_ISL_3718794 | hCoV-19/Puerto Rico/PR-CDC-S545/2021 | North America / Puerto Rico | 2021-08-09 |
| EPI_ISL_3730704 | hCoV-19/Puerto Rico/PR-CDC-S547/2021 | North America / Puerto Rico | 2021-06-18 |
| EPI_ISL_3730705 | hCoV-19/Puerto Rico/PR-CDC-S548/2021 | North America / Puerto Rico | 2021-06-19 |
| EPI_ISL_434541 | hCoV-19/Puerto Rico/PR-CDC-S1/2020 | North America / Puerto Rico | 2020-03-23 |
| EPI_ISL_434542 | hCoV-19/Puerto Rico/PR-CDC-S2/2020 | North America / Puerto Rico | 2020-03-23 |
| EPI_ISL_434543 | hCoV-19/Puerto Rico/PR-CDC-S3/2020 | North America / Puerto Rico | 2020-03-23 |
| EPI_ISL_434544 | hCoV-19/Puerto Rico/PR-CDC-S4/2020 | North America / Puerto Rico | 2020-03-23 |
| EPI_ISL_434545 | hCoV-19/Puerto Rico/PR-CDC-S5/2020 | North America / Puerto Rico | 2020-03-23 |
| EPI_ISL_434546 | hCoV-19/Puerto Rico/PR-CDC-S6/2020 | North America / Puerto Rico | 2020-03-24 |
| EPI_ISL_434547 | hCoV-19/Puerto Rico/PR-CDC-S7/2020 | North America / Puerto Rico | 2020-03-24 |
| EPI_ISL_434548 | hCoV-19/Puerto Rico/PR-CDC-S8/2020 | North America / Puerto Rico | 2020-03-23 |
| EPI_ISL_434549 | hCoV-19/Puerto Rico/PR-CDC-S9/2020 | North America / Puerto Rico | 2020-03-23 |
| EPI_ISL_434550 | hCoV-19/Puerto Rico/PR-CDC-S10/2020 | North America / Puerto Rico | 2020-03-24 |
| EPI_ISL_434551 | hCoV-19/Puerto Rico/PR-CDC-S11/2020 | North America / Puerto Rico | 2020-03-24 |
| EPI_ISL_434552 | hCoV-19/Puerto Rico/PR-CDC-S18/2020 | North America / Puerto Rico | 2020-04-01 |
| EPI_ISL_434553 | hCoV-19/Puerto Rico/PR-CDC-S19/2020 | North America / Puerto Rico | 2020-04-01 |
| EPI_ISL_5095325 | hCoV-19/Puerto Rico/PR-CDC-S598/2021 | North America / Puerto Rico | 2021-08-04 |
| EPI_ISL_5095326 | hCoV-19/Puerto Rico/PR-CDC-S599/2021 | North America / Puerto Rico | 2021-08-04 |
| EPI_ISL_5095327 | hCoV-19/Puerto Rico/PR-CDC-S600/2021 | North America / Puerto Rico | 2021-08-04 |
| EPI_ISL_5095328 | hCoV-19/Puerto Rico/PR-CDC-S601/2021 | North America / Puerto Rico | 2021-08-04 |
| EPI_ISL_5095329 | hCoV-19/Puerto Rico/PR-CDC-S602/2021 | North America / Puerto Rico | 2021-08-04 |
| EPI_ISL_5095330 | hCoV-19/Puerto Rico/PR-CDC-S603/2021 | North America / Puerto Rico | 2021-08-04 |
| EPI_ISL_5095331 | hCoV-19/Puerto Rico/PR-CDC-S604/2021 | North America / Puerto Rico | 2021-08-04 |
| EPI_ISL_5095332 | hCoV-19/Puerto Rico/PR-CDC-S605/2021 | North America / Puerto Rico | 2021-08-04 |
| EPI_ISL_5095333 | hCoV-19/Puerto Rico/PR-CDC-S606/2021 | North America / Puerto Rico | 2021-08-04 |
| EPI_ISL_5095334 | hCoV-19/Puerto Rico/PR-CDC-S607/2021 | North America / Puerto Rico | 2021-08-04 |
| EPI_ISL_5095335 | hCoV-19/Puerto Rico/PR-CDC-S608/2021 | North America / Puerto Rico | 2021-08-03 |
| EPI_ISL_5095337 | hCoV-19/Puerto Rico/PR-CDC-S609/2021 | North America / Puerto Rico | 2021-08-03 |
| EPI_ISL_5095338 | hCoV-19/Puerto Rico/PR-CDC-S610/2021 | North America / Puerto Rico | 2021-08-04 |
| EPI_ISL_5095339 | hCoV-19/Puerto Rico/PR-CDC-S611/2021 | North America / Puerto Rico | 2021-08-03 |
| EPI_ISL_5095340 | hCoV-19/Puerto Rico/PR-CDC-S612/2021 | North America / Puerto Rico | 2021-08-04 |
| EPI_ISL_5095341 | hCoV-19/Puerto Rico/PR-CDC-S613/2021 | North America / Puerto Rico | 2021-08-04 |
| EPI_ISL_5095342 | hCoV-19/Puerto Rico/PR-CDC-S614/2021 | North America / Puerto Rico | 2021-08-04 |
| EPI_ISL_5095343 | hCoV-19/Puerto Rico/PR-CDC-S615/2021 | North America / Puerto Rico | 2021-08-03 |
| EPI_ISL_5095344 | hCoV-19/Puerto Rico/PR-CDC-S616/2021 | North America / Puerto Rico | 2021-08-03 |
| EPI_ISL_5095345 | hCoV-19/Puerto Rico/PR-CDC-S617/2021 | North America / Puerto Rico | 2021-08-04 |
| EPI_ISL_5095346 | hCoV-19/Puerto Rico/PR-CDC-S618/2021 | North America / Puerto Rico | 2021-08-05 |
| EPI_ISL_5095347 | hCoV-19/Puerto Rico/PR-CDC-S619/2021 | North America / Puerto Rico | 2021-08-04 |
| EPI_ISL_5095348 | hCoV-19/Puerto Rico/PR-CDC-S620/2021 | North America / Puerto Rico | 2021-08-04 |
| EPI_ISL_5095349 | hCoV-19/Puerto Rico/PR-CDC-S621/2021 | North America / Puerto Rico | 2021-08-04 |
| EPI_ISL_5095350 | hCoV-19/Puerto Rico/PR-CDC-S622/2021 | North America / Puerto Rico | 2021-08-06 |
| EPI_ISL_5095351 | hCoV-19/Puerto Rico/PR-CDC-S623/2021 | North America / Puerto Rico | 2021-08-09 |
| EPI_ISL_5095352 | hCoV-19/Puerto Rico/PR-CDC-S624/2021 | North America / Puerto Rico | 2021-08-17 |
| EPI_ISL_5095353 | hCoV-19/Puerto Rico/PR-CDC-S625/2021 | North America / Puerto Rico | 2021-08-17 |
| EPI_ISL_5095354 | hCoV-19/Puerto Rico/PR-CDC-S626/2021 | North America / Puerto Rico | 2021-08-18 |
| EPI_ISL_5095355 | hCoV-19/Puerto Rico/PR-CDC-S627/2021 | North America / Puerto Rico | 2021-08-18 |
| EPI_ISL_5095356 | hCoV-19/Puerto Rico/PR-CDC-S628/2021 | North America / Puerto Rico | 2021-08-30 |
| EPI_ISL_5095357 | hCoV-19/Puerto Rico/PR-CDC-S629/2021 | North America / Puerto Rico | 2021-08-31 |
| EPI_ISL_5095358 | hCoV-19/Puerto Rico/PR-CDC-S630/2021 | North America / Puerto Rico | 2021-08-30 |
| EPI_ISL_5095359 | hCoV-19/Puerto Rico/PR-CDC-S631/2021 | North America / Puerto Rico | 2021-08-30 |
| EPI_ISL_5095360 | hCoV-19/Puerto Rico/PR-CDC-S632/2021 | North America / Puerto Rico | 2021-08-30 |
| EPI_ISL_5095361 | hCoV-19/Puerto Rico/PR-CDC-S633/2021 | North America / Puerto Rico | 2021-08-31 |
| EPI_ISL_5095362 | hCoV-19/Puerto Rico/PR-CDC-S634/2021 | North America / Puerto Rico | 2021-09-01 |
| EPI_ISL_5095363 | hCoV-19/Puerto Rico/PR-CDC-S635/2021 | North America / Puerto Rico | 2021-09-01 |
| EPI_ISL_5095364 | hCoV-19/Puerto Rico/PR-CDC-S636/2021 | North America / Puerto Rico | 2021-09-02 |
| EPI_ISL_5095365 | hCoV-19/Puerto Rico/PR-CDC-S637/2021 | North America / Puerto Rico | 2021-09-01 |
| EPI_ISL_5095366 | hCoV-19/Puerto Rico/PR-CDC-S638/2021 | North America / Puerto Rico | 2021-09-02 |
| EPI_ISL_5095367 | hCoV-19/Puerto Rico/PR-CDC-S639/2021 | North America / Puerto Rico | 2021-09-02 |
| EPI_ISL_5095368 | hCoV-19/Puerto Rico/PR-CDC-S640/2021 | North America / Puerto Rico | 2021-09-02 |
| EPI_ISL_5095369 | hCoV-19/Puerto Rico/PR-CDC-S641/2021 | North America / Puerto Rico | 2021-09-01 |
| EPI_ISL_5095370 | hCoV-19/Puerto Rico/PR-CDC-S642/2021 | North America / Puerto Rico | 2021-09-02 |
| EPI_ISL_5095371 | hCoV-19/Puerto Rico/PR-CDC-S643/2021 | North America / Puerto Rico | 2021-09-02 |
| EPI_ISL_5095372 | hCoV-19/Puerto Rico/PR-CDC-S644/2021 | North America / Puerto Rico | 2021-09-07 |
| EPI_ISL_5095373 | hCoV-19/Puerto Rico/PR-CDC-S645/2021 | North America / Puerto Rico | 2021-09-07 |
| EPI_ISL_5095374 | hCoV-19/Puerto Rico/PR-CDC-S646/2021 | North America / Puerto Rico | 2021-09-08 |
| EPI_ISL_5095375 | hCoV-19/Puerto Rico/PR-CDC-S647/2021 | North America / Puerto Rico | 2021-09-09 |
| EPI_ISL_5095376 | hCoV-19/Puerto Rico/PR-CDC-S648/2021 | North America / Puerto Rico | 2021-09-09 |
| EPI_ISL_5095377 | hCoV-19/Puerto Rico/PR-CDC-S649/2021 | North America / Puerto Rico | 2021-09-09 |
| EPI_ISL_5095378 | hCoV-19/Puerto Rico/PR-CDC-S650/2021 | North America / Puerto Rico | 2021-09-09 |
| EPI_ISL_5095379 | hCoV-19/Puerto Rico/PR-CDC-S651/2021 | North America / Puerto Rico | 2021-09-09 |
| EPI_ISL_5095380 | hCoV-19/Puerto Rico/PR-CDC-S652/2021 | North America / Puerto Rico | 2021-09-09 |
| EPI_ISL_5095381 | hCoV-19/Puerto Rico/PR-CDC-S653/2021 | North America / Puerto Rico | 2021-09-09 |
| EPI_ISL_5095382 | hCoV-19/Puerto Rico/PR-CDC-S654/2021 | North America / Puerto Rico | 2021-09-09 |
| EPI_ISL_5095383 | hCoV-19/Puerto Rico/PR-CDC-S655/2021 | North America / Puerto Rico | 2021-09-08 |
| EPI_ISL_5095384 | hCoV-19/Puerto Rico/PR-CDC-S656/2021 | North America / Puerto Rico | 2021-09-13 |
| EPI_ISL_5095385 | hCoV-19/Puerto Rico/PR-CDC-S657/2021 | North America / Puerto Rico | 2021-09-13 |
| EPI_ISL_5095386 | hCoV-19/Puerto Rico/PR-CDC-S658/2021 | North America / Puerto Rico | 2021-09-13 |
| EPI_ISL_5095387 | hCoV-19/Puerto Rico/PR-CDC-S659/2021 | North America / Puerto Rico | 2021-07-21 |
| EPI_ISL_5095388 | hCoV-19/Puerto Rico/PR-CDC-S661/2021 | North America / Puerto Rico | 2021-07-27 |
| EPI_ISL_5095389 | hCoV-19/Puerto Rico/PR-CDC-S662/2021 | North America / Puerto Rico | 2021-07-27 |
| EPI_ISL_5095390 | hCoV-19/Puerto Rico/PR-CDC-S663/2021 | North America / Puerto Rico | 2021-08-04 |
| EPI_ISL_5095391 | hCoV-19/Puerto Rico/PR-CDC-S664/2021 | North America / Puerto Rico | 2021-07-30 |
| EPI_ISL_5095392 | hCoV-19/Puerto Rico/PR-CDC-S665/2021 | North America / Puerto Rico | 2021-08-02 |
| EPI_ISL_5095393 | hCoV-19/Puerto Rico/PR-CDC-S666/2021 | North America / Puerto Rico | 2021-08-03 |
| EPI_ISL_5095394 | hCoV-19/Puerto Rico/PR-CDC-S667/2021 | North America / Puerto Rico | 2021-08-03 |
| EPI_ISL_5095395 | hCoV-19/Puerto Rico/PR-CDC-S668/2021 | North America / Puerto Rico | 2021-08-04 |
| EPI_ISL_5095396 | hCoV-19/Puerto Rico/PR-CDC-S669/2021 | North America / Puerto Rico | 2021-08-16 |
| EPI_ISL_5095397 | hCoV-19/Puerto Rico/PR-CDC-S670/2021 | North America / Puerto Rico | 2021-08-17 |
| EPI_ISL_5095398 | hCoV-19/Puerto Rico/PR-CDC-S671/2021 | North America / Puerto Rico | 2021-08-16 |
| EPI_ISL_5095399 | hCoV-19/Puerto Rico/PR-CDC-S672/2021 | North America / Puerto Rico | 2021-08-16 |
| EPI_ISL_5095400 | hCoV-19/Puerto Rico/PR-CDC-S673/2021 | North America / Puerto Rico | 2021-08-16 |
| EPI_ISL_5095401 | hCoV-19/Puerto Rico/PR-CDC-S674/2021 | North America / Puerto Rico | 2021-08-17 |
| EPI_ISL_5095402 | hCoV-19/Puerto Rico/PR-CDC-S675/2021 | North America / Puerto Rico | 2021-08-17 |
| EPI_ISL_5095403 | hCoV-19/Puerto Rico/PR-CDC-S676/2021 | North America / Puerto Rico | 2021-08-12 |
| EPI_ISL_5095404 | hCoV-19/Puerto Rico/PR-CDC-S677/2021 | North America / Puerto Rico | 2021-08-13 |
| EPI_ISL_5095405 | hCoV-19/Puerto Rico/PR-CDC-S678/2021 | North America / Puerto Rico | 2021-08-13 |
| EPI_ISL_5095406 | hCoV-19/Puerto Rico/PR-CDC-S679/2021 | North America / Puerto Rico | 2021-08-15 |
| EPI_ISL_5095407 | hCoV-19/Puerto Rico/PR-CDC-S680/2021 | North America / Puerto Rico | 2021-08-11 |
| EPI_ISL_5095408 | hCoV-19/Puerto Rico/PR-CDC-S681/2021 | North America / Puerto Rico | 2021-08-12 |
| EPI_ISL_5095409 | hCoV-19/Puerto Rico/PR-CDC-S682/2021 | North America / Puerto Rico | 2021-08-12 |
| EPI_ISL_5095410 | hCoV-19/Puerto Rico/PR-CDC-S683/2021 | North America / Puerto Rico | 2021-08-12 |
| EPI_ISL_5095411 | hCoV-19/Puerto Rico/PR-CDC-S684/2021 | North America / Puerto Rico | 2021-08-11 |
| EPI_ISL_5104527 | hCoV-19/Puerto Rico/PR-CDC-S660/2021 | North America / Puerto Rico | 2021-07-21 |
| EPI_ISL_539495 | hCoV-19/Puerto Rico/PR-CDC-S29/2020 | North America / Puerto Rico | 2020-07-06 |
| EPI_ISL_5781943 | hCoV-19/Puerto Rico/PR-CDC-S685/2021 | North America / Puerto Rico | 2021-09-22 |
| EPI_ISL_5781944 | hCoV-19/Puerto Rico/PR-CDC-S687/2021 | North America / Puerto Rico | 2021-09-26 |
| EPI_ISL_5781945 | hCoV-19/Puerto Rico/PR-CDC-S688/2021 | North America / Puerto Rico | 2021-08-18 |
| EPI_ISL_5781946 | hCoV-19/Puerto Rico/PR-CDC-S689/2021 | North America / Puerto Rico | 2021-08-18 |
| EPI_ISL_5781947 | hCoV-19/Puerto Rico/PR-CDC-S690/2021 | North America / Puerto Rico | 2021-08-18 |
| EPI_ISL_5781948 | hCoV-19/Puerto Rico/PR-CDC-S691/2021 | North America / Puerto Rico | 2021-08-21 |
| EPI_ISL_5781949 | hCoV-19/Puerto Rico/PR-CDC-S692/2021 | North America / Puerto Rico | 2021-08-19 |
| EPI_ISL_5781950 | hCoV-19/Puerto Rico/PR-CDC-S693/2021 | North America / Puerto Rico | 2021-08-20 |
| EPI_ISL_5781951 | hCoV-19/Puerto Rico/PR-CDC-S694/2021 | North America / Puerto Rico | 2021-08-24 |
| EPI_ISL_5781952 | hCoV-19/Puerto Rico/PR-CDC-S695/2021 | North America / Puerto Rico | 2021-08-23 |
| EPI_ISL_5781953 | hCoV-19/Puerto Rico/PR-CDC-S696/2021 | North America / Puerto Rico | 2021-08-24 |
| EPI_ISL_5781954 | hCoV-19/Puerto Rico/PR-CDC-S697/2021 | North America / Puerto Rico | 2021-08-18 |
| EPI_ISL_5781955 | hCoV-19/Puerto Rico/PR-CDC-S698/2021 | North America / Puerto Rico | 2021-08-28 |
| EPI_ISL_5781956 | hCoV-19/Puerto Rico/PR-CDC-S699/2021 | North America / Puerto Rico | 2021-08-26 |
| EPI_ISL_5781957 | hCoV-19/Puerto Rico/PR-CDC-S700/2021 | North America / Puerto Rico | 2021-08-28 |
| EPI_ISL_5781958 | hCoV-19/Puerto Rico/PR-CDC-S701/2021 | North America / Puerto Rico | 2021-08-26 |
| EPI_ISL_5781959 | hCoV-19/Puerto Rico/PR-CDC-S702/2021 | North America / Puerto Rico | 2021-08-27 |
| EPI_ISL_5781960 | hCoV-19/Puerto Rico/PR-CDC-S703/2021 | North America / Puerto Rico | 2021-08-28 |
| EPI_ISL_5781961 | hCoV-19/Puerto Rico/PR-CDC-S704/2021 | North America / Puerto Rico | 2021-08-20 |
| EPI_ISL_5781962 | hCoV-19/Puerto Rico/PR-CDC-S705/2021 | North America / Puerto Rico | 2021-08-28 |
| EPI_ISL_5781963 | hCoV-19/Puerto Rico/PR-CDC-S706/2021 | North America / Puerto Rico | 2021-08-27 |
| EPI_ISL_5781964 | hCoV-19/Puerto Rico/PR-CDC-S707/2021 | North America / Puerto Rico | 2021-08-28 |
| EPI_ISL_5781965 | hCoV-19/Puerto Rico/PR-CDC-S709/2021 | North America / Puerto Rico | 2021-08-25 |
| EPI_ISL_5781966 | hCoV-19/Puerto Rico/PR-CDC-S710/2021 | North America / Puerto Rico | 2021-09-07 |
| EPI_ISL_5781967 | hCoV-19/Puerto Rico/PR-CDC-S711/2021 | North America / Puerto Rico | 2021-09-04 |
| EPI_ISL_5781968 | hCoV-19/Puerto Rico/PR-CDC-S712/2021 | North America / Puerto Rico | 2021-09-04 |
| EPI_ISL_5781969 | hCoV-19/Puerto Rico/PR-CDC-S713/2021 | North America / Puerto Rico | 2021-09-09 |
| EPI_ISL_5781970 | hCoV-19/Puerto Rico/PR-CDC-S714/2021 | North America / Puerto Rico | 2021-09-14 |
| EPI_ISL_5781971 | hCoV-19/Puerto Rico/PR-CDC-S715/2021 | North America / Puerto Rico | 2021-09-12 |
| EPI_ISL_5781972 | hCoV-19/Puerto Rico/PR-CDC-S716/2021 | North America / Puerto Rico | 2021-09-13 |
| EPI_ISL_5781973 | hCoV-19/Puerto Rico/PR-CDC-S717/2021 | North America / Puerto Rico | 2021-08-30 |
| EPI_ISL_5781974 | hCoV-19/Puerto Rico/PR-CDC-S718/2021 | North America / Puerto Rico | 2021-08-30 |
| EPI_ISL_5781975 | hCoV-19/Puerto Rico/PR-CDC-S719/2021 | North America / Puerto Rico | 2021-08-30 |
| EPI_ISL_5781976 | hCoV-19/Puerto Rico/PR-CDC-S720/2021 | North America / Puerto Rico | 2021-09-18 |
| EPI_ISL_5781977 | hCoV-19/Puerto Rico/PR-CDC-S721/2021 | North America / Puerto Rico | 2021-09-15 |
| EPI_ISL_5781978 | hCoV-19/Puerto Rico/PR-CDC-S722/2021 | North America / Puerto Rico | 2021-09-17 |
| EPI_ISL_5781979 | hCoV-19/Puerto Rico/PR-CDC-S723/2021 | North America / Puerto Rico | 2021-09-20 |
| EPI_ISL_5781980 | hCoV-19/Puerto Rico/PR-CDC-S724/2021 | North America / Puerto Rico | 2021-09-22 |
| EPI_ISL_5781981 | hCoV-19/Puerto Rico/PR-CDC-S725/2021 | North America / Puerto Rico | 2021-09-21 |
| EPI_ISL_5781982 | hCoV-19/Puerto Rico/PR-CDC-S726/2021 | North America / Puerto Rico | 2021-09-22 |
| EPI_ISL_5781983 | hCoV-19/Puerto Rico/PR-CDC-S728/2021 | North America / Puerto Rico | 2021-09-22 |
| EPI_ISL_5781984 | hCoV-19/Puerto Rico/PR-CDC-S729/2021 | North America / Puerto Rico | 2021-09-23 |
| EPI_ISL_5781985 | hCoV-19/Puerto Rico/PR-CDC-S730/2021 | North America / Puerto Rico | 2021-09-23 |
| EPI_ISL_5781986 | hCoV-19/Puerto Rico/PR-CDC-S731/2021 | North America / Puerto Rico | 2021-09-23 |
| EPI_ISL_5781987 | hCoV-19/Puerto Rico/PR-CDC-S732/2021 | North America / Puerto Rico | 2021-09-23 |
| EPI_ISL_5781988 | hCoV-19/Puerto Rico/PR-CDC-S733/2021 | North America / Puerto Rico | 2021-09-23 |
| EPI_ISL_5781989 | hCoV-19/Puerto Rico/PR-CDC-S734/2021 | North America / Puerto Rico | 2021-09-23 |
| EPI_ISL_5781990 | hCoV-19/Puerto Rico/PR-CDC-S735/2021 | North America / Puerto Rico | 2021-09-23 |
| EPI_ISL_5781991 | hCoV-19/Puerto Rico/PR-CDC-S736/2021 | North America / Puerto Rico | 2021-09-24 |
| EPI_ISL_5781992 | hCoV-19/Puerto Rico/PR-CDC-S737/2021 | North America / Puerto Rico | 2021-09-24 |
| EPI_ISL_5781993 | hCoV-19/Puerto Rico/PR-CDC-S738/2021 | North America / Puerto Rico | 2021-09-24 |
| EPI_ISL_5781994 | hCoV-19/Puerto Rico/PR-CDC-S739/2021 | North America / Puerto Rico | 2021-09-24 |
| EPI_ISL_5781995 | hCoV-19/Puerto Rico/PR-CDC-S740/2021 | North America / Puerto Rico | 2021-09-24 |
| EPI_ISL_5781996 | hCoV-19/Puerto Rico/PR-CDC-S741/2021 | North America / Puerto Rico | 2021-09-24 |
| EPI_ISL_5781997 | hCoV-19/Puerto Rico/PR-CDC-S742/2021 | North America / Puerto Rico | 2021-09-27 |
| EPI_ISL_5781998 | hCoV-19/Puerto Rico/PR-CDC-S743/2021 | North America / Puerto Rico | 2021-09-27 |
| EPI_ISL_5781999 | hCoV-19/Puerto Rico/PR-CDC-S744/2021 | North America / Puerto Rico | 2021-09-27 |
| EPI_ISL_5782000 | hCoV-19/Puerto Rico/PR-CDC-S745/2021 | North America / Puerto Rico | 2021-09-27 |
| EPI_ISL_5782001 | hCoV-19/Puerto Rico/PR-CDC-S747/2021 | North America / Puerto Rico | 2021-09-28 |
| EPI_ISL_5782002 | hCoV-19/Puerto Rico/PR-CDC-S748/2021 | North America / Puerto Rico | 2021-09-28 |
| EPI_ISL_5782003 | hCoV-19/Puerto Rico/PR-CDC-S749/2021 | North America / Puerto Rico | 2021-09-28 |
| EPI_ISL_5782004 | hCoV-19/Puerto Rico/PR-CDC-S750/2021 | North America / Puerto Rico | 2021-09-28 |
| EPI_ISL_5782005 | hCoV-19/Puerto Rico/PR-CDC-S751/2021 | North America / Puerto Rico | 2021-09-28 |
| EPI_ISL_5782006 | hCoV-19/Puerto Rico/PR-CDC-S752/2021 | North America / Puerto Rico | 2021-09-29 |
| EPI_ISL_5782007 | hCoV-19/Puerto Rico/PR-CDC-S753/2021 | North America / Puerto Rico | 2021-09-29 |
| EPI_ISL_5782008 | hCoV-19/Puerto Rico/PR-CDC-S754/2021 | North America / Puerto Rico | 2021-09-29 |
| EPI_ISL_5782009 | hCoV-19/Puerto Rico/PR-CDC-S755/2021 | North America / Puerto Rico | 2021-09-29 |
| EPI_ISL_5782010 | hCoV-19/Puerto Rico/PR-CDC-S756/2021 | North America / Puerto Rico | 2021-09-29 |
| EPI_ISL_5782011 | hCoV-19/Puerto Rico/PR-CDC-S757/2021 | North America / Puerto Rico | 2021-09-29 |
| EPI_ISL_5782012 | hCoV-19/Puerto Rico/PR-CDC-S758/2021 | North America / Puerto Rico | 2021-09-29 |
| EPI_ISL_5782013 | hCoV-19/Puerto Rico/PR-CDC-S759/2021 | North America / Puerto Rico | 2021-09-29 |
| EPI_ISL_5782014 | hCoV-19/Puerto Rico/PR-CDC-S760/2021 | North America / Puerto Rico | 2021-09-29 |
| EPI_ISL_5782015 | hCoV-19/Puerto Rico/PR-CDC-S761/2021 | North America / Puerto Rico | 2021-09-29 |
| EPI_ISL_5782016 | hCoV-19/Puerto Rico/PR-CDC-S762/2021 | North America / Puerto Rico | 2021-09-02 |
| EPI_ISL_5782017 | hCoV-19/Puerto Rico/PR-CDC-S763/2021 | North America / Puerto Rico | 2021-09-02 |
| EPI_ISL_5782018 | hCoV-19/Puerto Rico/PR-CDC-S764/2021 | North America / Puerto Rico | 2021-09-02 |
| EPI_ISL_5782019 | hCoV-19/Puerto Rico/PR-CDC-S765/2021 | North America / Puerto Rico | 2021-09-02 |
| EPI_ISL_5782020 | hCoV-19/Puerto Rico/PR-CDC-S766/2021 | North America / Puerto Rico | 2021-09-02 |
| EPI_ISL_5782021 | hCoV-19/Puerto Rico/PR-CDC-S767/2021 | North America / Puerto Rico | 2021-09-02 |
| EPI_ISL_5782022 | hCoV-19/Puerto Rico/PR-CDC-S768/2021 | North America / Puerto Rico | 2021-09-07 |
| EPI_ISL_5782023 | hCoV-19/Puerto Rico/PR-CDC-S769/2021 | North America / Puerto Rico | 2021-09-07 |
| EPI_ISL_5782024 | hCoV-19/Puerto Rico/PR-CDC-S770/2021 | North America / Puerto Rico | 2021-09-07 |
| EPI_ISL_5782025 | hCoV-19/Puerto Rico/PR-CDC-S771/2021 | North America / Puerto Rico | 2021-09-07 |
| EPI_ISL_5845389 | hCoV-19/Puerto Rico/PR-CDC-S686/2021 | North America / Puerto Rico | 2021-09-23 |
| EPI_ISL_5845390 | hCoV-19/Puerto Rico/PR-CDC-S708/2021 | North America / Puerto Rico | 2021-08-27 |
| EPI_ISL_5845391 | hCoV-19/Puerto Rico/PR-CDC-S727/2021 | North America / Puerto Rico | 2021-09-22 |
| EPI_ISL_5845392 | hCoV-19/Puerto Rico/PR-CDC-S746/2021 | North America / Puerto Rico | 2021-09-27 |
| EPI_ISL_940756 | hCoV-19/Puerto Rico/PR-CDC-S20/2020 | North America / Puerto Rico | 2020-08-06 |
| EPI_ISL_940895 | hCoV-19/Puerto Rico/PR-CDC-S21/2020 | North America / Puerto Rico | 2020-08-08 |
| EPI_ISL_940896 | hCoV-19/Puerto Rico/PR-CDC-S22/2020 | North America / Puerto Rico | 2020-08-06 |
| EPI_ISL_940897 | hCoV-19/Puerto Rico/PR-CDC-S23/2020 | North America / Puerto Rico | 2020-08-06 |
| EPI_ISL_940898 | hCoV-19/Puerto Rico/PR-CDC-S24/2020 | North America / Puerto Rico | 2020-08-11 |
| EPI_ISL_940899 | hCoV-19/Puerto Rico/PR-CDC-S25/2020 | North America / Puerto Rico | 2020-08-10 |
| EPI_ISL_940900 | hCoV-19/Puerto Rico/PR-CDC-S26/2020 | North America / Puerto Rico | 2020-06-29 |
| EPI_ISL_940901 | hCoV-19/Puerto Rico/PR-CDC-S27/2020 | North America / Puerto Rico | 2020-06-27 |
| EPI_ISL_940902 | hCoV-19/Puerto Rico/PR-CDC-S28/2020 | North America / Puerto Rico | 2020-07-15 |
| EPI_ISL_940903 | hCoV-19/Puerto Rico/PR-CDC-S30/2020 | North America / Puerto Rico | 2020-06-11 |
| EPI_ISL_940904 | hCoV-19/Puerto Rico/PR-CDC-S31/2020 | North America / Puerto Rico | 2020-06-11 |
| EPI_ISL_940905 | hCoV-19/Puerto Rico/PR-CDC-S32/2020 | North America / Puerto Rico | 2020-07-07 |
| EPI_ISL_940906 | hCoV-19/Puerto Rico/PR-CDC-S33/2020 | North America / Puerto Rico | 2020-07-14 |
| EPI_ISL_940907 | hCoV-19/Puerto Rico/PR-CDC-S34/2020 | North America / Puerto Rico | 2020-06-11 |
| EPI_ISL_940908 | hCoV-19/Puerto Rico/PR-CDC-S35/2020 | North America / Puerto Rico | 2020-07-09 |
| EPI_ISL_940909 | hCoV-19/Puerto Rico/PR-CDC-S36/2020 | North America / Puerto Rico | 2020-07-14 |
| EPI_ISL_940910 | hCoV-19/Puerto Rico/PR-CDC-S37/2020 | North America / Puerto Rico | 2020-07-14 |
| EPI_ISL_940911 | hCoV-19/Puerto Rico/PR-CDC-S38/2020 | North America / Puerto Rico | 2020-07-14 |
| EPI_ISL_940912 | hCoV-19/Puerto Rico/PR-CDC-S39/2020 | North America / Puerto Rico | 2020-06-22 |
| EPI_ISL_940913 | hCoV-19/Puerto Rico/PR-CDC-S40/2020 | North America / Puerto Rico | 2020-07-17 |
| EPI_ISL_940914 | hCoV-19/Puerto Rico/PR-CDC-S41/2020 | North America / Puerto Rico | 2020-07-15 |
| EPI_ISL_940915 | hCoV-19/Puerto Rico/PR-CDC-S42/2020 | North America / Puerto Rico | 2020-07-21 |
| EPI_ISL_940916 | hCoV-19/Puerto Rico/PR-CDC-S43/2020 | North America / Puerto Rico | 2020-07-15 |
| EPI_ISL_940917 | hCoV-19/Puerto Rico/PR-CDC-S44/2020 | North America / Puerto Rico | 2020-07-20 |
| EPI_ISL_940918 | hCoV-19/Puerto Rico/PR-CDC-S45/2020 | North America / Puerto Rico | 2020-07-20 |
| EPI_ISL_940919 | hCoV-19/Puerto Rico/PR-CDC-S46/2020 | North America / Puerto Rico | 2020-07-15 |
| EPI_ISL_940920 | hCoV-19/Puerto Rico/PR-CDC-S47/2020 | North America / Puerto Rico | 2020-07-24 |
| EPI_ISL_940921 | hCoV-19/Puerto Rico/PR-CDC-S48/2020 | North America / Puerto Rico | 2020-07-27 |
| EPI_ISL_940922 | hCoV-19/Puerto Rico/PR-CDC-S49/2020 | North America / Puerto Rico | 2020-07-23 |
| EPI_ISL_940923 | hCoV-19/Puerto Rico/PR-CDC-S50/2020 | North America / Puerto Rico | 2020-07-23 |
| EPI_ISL_940924 | hCoV-19/Puerto Rico/PR-CDC-S51/2020 | North America / Puerto Rico | 2020-07-28 |
| EPI_ISL_940925 | hCoV-19/Puerto Rico/PR-CDC-S52/2020 | North America / Puerto Rico | 2020-07-27 |
| EPI_ISL_940926 | hCoV-19/Puerto Rico/PR-CDC-S53/2020 | North America / Puerto Rico | 2020-08-03 |
| EPI_ISL_940927 | hCoV-19/Puerto Rico/PR-CDC-S54/2020 | North America / Puerto Rico | 2020-07-23 |
| EPI_ISL_940928 | hCoV-19/Puerto Rico/PR-CDC-S55/2020 | North America / Puerto Rico | 2020-07-22 |
| EPI_ISL_940929 | hCoV-19/Puerto Rico/PR-CDC-S56/2020 | North America / Puerto Rico | 2020-08-03 |
| EPI_ISL_940930 | hCoV-19/Puerto Rico/PR-CDC-S57/2020 | North America / Puerto Rico | 2020-08-03 |
| EPI_ISL_940931 | hCoV-19/Puerto Rico/PR-CDC-S58/2020 | North America / Puerto Rico | 2020-08-03 |
| EPI_ISL_940932 | hCoV-19/Puerto Rico/PR-CDC-S59/2020 | North America / Puerto Rico | 2020-08-02 |
| EPI_ISL_940933 | hCoV-19/Puerto Rico/PR-CDC-S60/2020 | North America / Puerto Rico | 2020-07-31 |
| EPI_ISL_940934 | hCoV-19/Puerto Rico/PR-CDC-S61/2020 | North America / Puerto Rico | 2020-08-01 |
| EPI_ISL_940935 | hCoV-19/Puerto Rico/PR-CDC-S62/2020 | North America / Puerto Rico | 2020-08-03 |
| EPI_ISL_940936 | hCoV-19/Puerto Rico/PR-CDC-S63/2020 | North America / Puerto Rico | 2020-10-15 |
| EPI_ISL_940937 | hCoV-19/Puerto Rico/PR-CDC-S64/2020 | North America / Puerto Rico | 2020-10-14 |
| EPI_ISL_940938 | hCoV-19/Puerto Rico/PR-CDC-S65/2020 | North America / Puerto Rico | 2020-10-14 |
| EPI_ISL_940939 | hCoV-19/Puerto Rico/PR-CDC-S66/2020 | North America / Puerto Rico | 2020-10-18 |
| EPI_ISL_940940 | hCoV-19/Puerto Rico/PR-CDC-S67/2020 | North America / Puerto Rico | 2020-10-18 |
| EPI_ISL_940941 | hCoV-19/Puerto Rico/PR-CDC-S68/2020 | North America / Puerto Rico | 2020-10-18 |
| EPI_ISL_940942 | hCoV-19/Puerto Rico/PR-CDC-S69/2020 | North America / Puerto Rico | 2020-10-20 |
| EPI_ISL_940943 | hCoV-19/Puerto Rico/PR-CDC-S70/2020 | North America / Puerto Rico | 2020-10-19 |
| EPI_ISL_940944 | hCoV-19/Puerto Rico/PR-CDC-S71/2020 | North America / Puerto Rico | 2020-10-20 |
| EPI_ISL_940945 | hCoV-19/Puerto Rico/PR-CDC-S72/2020 | North America / Puerto Rico | 2020-10-20 |
| EPI_ISL_940946 | hCoV-19/Puerto Rico/PR-CDC-S73/2020 | North America / Puerto Rico | 2020-10-20 |
| EPI_ISL_940947 | hCoV-19/Puerto Rico/PR-CDC-S74/2020 | North America / Puerto Rico | 2020-10-19 |
| EPI_ISL_940948 | hCoV-19/Puerto Rico/PR-CDC-S75/2020 | North America / Puerto Rico | 2020-10-19 |
| EPI_ISL_940949 | hCoV-19/Puerto Rico/PR-CDC-S76/2020 | North America / Puerto Rico | 2020-10-19 |
| EPI_ISL_940950 | hCoV-19/Puerto Rico/PR-CDC-S77/2020 | North America / Puerto Rico | 2020-08-23 |
| EPI_ISL_940951 | hCoV-19/Puerto Rico/PR-CDC-S78/2020 | North America / Puerto Rico | 2020-08-19 |
| EPI_ISL_940952 | hCoV-19/Puerto Rico/PR-CDC-S79/2020 | North America / Puerto Rico | 2020-08-20 |
| EPI_ISL_940953 | hCoV-19/Puerto Rico/PR-CDC-S80/2020 | North America / Puerto Rico | 2020-08-20 |
| EPI_ISL_940954 | hCoV-19/Puerto Rico/PR-CDC-S81/2020 | North America / Puerto Rico | 2020-09-01 |
| EPI_ISL_940955 | hCoV-19/Puerto Rico/PR-CDC-S82/2020 | North America / Puerto Rico | 2020-08-27 |
| EPI_ISL_940956 | hCoV-19/Puerto Rico/PR-CDC-S83/2020 | North America / Puerto Rico | 2020-08-27 |
| EPI_ISL_940957 | hCoV-19/Puerto Rico/PR-CDC-S84/2020 | North America / Puerto Rico | 2020-08-28 |
| EPI_ISL_940958 | hCoV-19/Puerto Rico/PR-CDC-S85/2020 | North America / Puerto Rico | 2020-08-28 |
| EPI_ISL_940959 | hCoV-19/Puerto Rico/PR-CDC-S86/2020 | North America / Puerto Rico | 2020-08-25 |
| EPI_ISL_940960 | hCoV-19/Puerto Rico/PR-CDC-S87/2020 | North America / Puerto Rico | 2020-09-27 |
| EPI_ISL_940961 | hCoV-19/Puerto Rico/PR-CDC-S88/2020 | North America / Puerto Rico | 2020-09-25 |
| EPI_ISL_940962 | hCoV-19/Puerto Rico/PR-CDC-S92/2020 | North America / Puerto Rico | 2020-09-18 |
| EPI_ISL_940963 | hCoV-19/Puerto Rico/PR-CDC-S94/2020 | North America / Puerto Rico | 2020-09-04 |
| EPI_ISL_940964 | hCoV-19/Puerto Rico/PR-CDC-S95/2020 | North America / Puerto Rico | 2020-09-05 |
| EPI_ISL_940965 | hCoV-19/Puerto Rico/PR-CDC-S96/2020 | North America / Puerto Rico | 2020-09-05 |
| EPI_ISL_940966 | hCoV-19/Puerto Rico/PR-CDC-S98/2020 | North America / Puerto Rico | 2020-09-05 |
| EPI_ISL_940967 | hCoV-19/Puerto Rico/PR-CDC-S99/2020 | North America / Puerto Rico | 2020-09-06 |
| EPI_ISL_940968 | hCoV-19/Puerto Rico/PR-CDC-S100/2020 | North America / Puerto Rico | 2020-09-02 |
| EPI_ISL_940969 | hCoV-19/Puerto Rico/PR-CDC-S101/2020 | North America / Puerto Rico | 2020-09-04 |
| EPI_ISL_940970 | hCoV-19/Puerto Rico/PR-CDC-S102/2020 | North America / Puerto Rico | 2020-09-04 |
| EPI_ISL_940971 | hCoV-19/Puerto Rico/PR-CDC-S103/2020 | North America / Puerto Rico | 2020-09-02 |
| EPI_ISL_940972 | hCoV-19/Puerto Rico/PR-CDC-S105/2020 | North America / Puerto Rico | 2020-09-02 |
| EPI_ISL_940973 | hCoV-19/Puerto Rico/PR-CDC-S106/2020 | North America / Puerto Rico | 2020-09-11 |
| EPI_ISL_940974 | hCoV-19/Puerto Rico/PR-CDC-S107/2020 | North America / Puerto Rico | 2020-09-14 |
| EPI_ISL_940975 | hCoV-19/Puerto Rico/PR-CDC-S108/2020 | North America / Puerto Rico | 2020-09-12 |
| EPI_ISL_940976 | hCoV-19/Puerto Rico/PR-CDC-S109/2020 | North America / Puerto Rico | 2020-10-08 |
| EPI_ISL_940977 | hCoV-19/Puerto Rico/PR-CDC-S110/2020 | North America / Puerto Rico | 2020-10-08 |
| EPI_ISL_940978 | hCoV-19/Puerto Rico/PR-CDC-S111/2020 | North America / Puerto Rico | 2020-10-07 |
| EPI_ISL_940979 | hCoV-19/Puerto Rico/PR-CDC-S112/2020 | North America / Puerto Rico | 2020-10-12 |
| EPI_ISL_940980 | hCoV-19/Puerto Rico/PR-CDC-S113/2020 | North America / Puerto Rico | 2020-10-13 |
| EPI_ISL_940981 | hCoV-19/Puerto Rico/PR-CDC-S114/2020 | North America / Puerto Rico | 2020-10-10 |
| EPI_ISL_940982 | hCoV-19/Puerto Rico/PR-CDC-S115/2020 | North America / Puerto Rico | 2020-10-10 |
| EPI_ISL_940983 | hCoV-19/Puerto Rico/PR-CDC-S116/2020 | North America / Puerto Rico | 2020-10-01 |
| EPI_ISL_940984 | hCoV-19/Puerto Rico/PR-CDC-S117/2020 | North America / Puerto Rico | 2020-10-02 |
| EPI_ISL_940985 | hCoV-19/Puerto Rico/PR-CDC-S118/2020 | North America / Puerto Rico | 2020-10-02 |
| EPI_ISL_940986 | hCoV-19/Puerto Rico/PR-CDC-S119/2020 | North America / Puerto Rico | 2020-09-30 |
| EPI_ISL_940987 | hCoV-19/Puerto Rico/PR-CDC-S120/2020 | North America / Puerto Rico | 2020-09-25 |
| EPI_ISL_940988 | hCoV-19/Puerto Rico/PR-CDC-S121/2020 | North America / Puerto Rico | 2020-10-03 |
| EPI_ISL_940989 | hCoV-19/Puerto Rico/PR-CDC-S122/2020 | North America / Puerto Rico | 2020-10-02 |
| EPI_ISL_940990 | hCoV-19/Puerto Rico/PR-CDC-S123/2020 | North America / Puerto Rico | 2020-10-02 |
| EPI_ISL_940991 | hCoV-19/Puerto Rico/PR-CDC-S124/2020 | North America / Puerto Rico | 2020-10-03 |
| EPI_ISL_940992 | hCoV-19/Puerto Rico/PR-CDC-S125/2020 | North America / Puerto Rico | 2020-10-02 |
| EPI_ISL_940993 | hCoV-19/Puerto Rico/PR-CDC-S126/2020 | North America / Puerto Rico | 2020-10-04 |
| EPI_ISL_940994 | hCoV-19/Puerto Rico/PR-CDC-S127/2020 | North America / Puerto Rico | 2020-10-21 |
| EPI_ISL_942007 | hCoV-19/Puerto Rico/PR-CDC-S89/2020 | North America / Puerto Rico | 2020-09-21 |
| EPI_ISL_942008 | hCoV-19/Puerto Rico/PR-CDC-S90/2020 | North America / Puerto Rico | 2020-09-27 |
| EPI_ISL_942009 | hCoV-19/Puerto Rico/PR-CDC-S91/2020 | North America / Puerto Rico | 2020-09-22 |
| EPI_ISL_942010 | hCoV-19/Puerto Rico/PR-CDC-S93/2020 | North America / Puerto Rico | 2020-09-16 |
| EPI_ISL_942011 | hCoV-19/Puerto Rico/PR-CDC-S97/2020 | North America / Puerto Rico | 2020-09-06 |
| EPI_ISL_942012 | hCoV-19/Puerto Rico/PR-CDC-S104/2020 | North America / Puerto Rico | 2020-09-02 |
