# Supplementary material for: Genomic surveillance of SARS-CoV-2 in Puerto Rico enabled early detection and tracking of variants
Source: Commun Med (Lond). 2022 Aug 11;2:100. doi: 10.1038/s43856-022-00168-7 (PMC9366129; doi:10.1038/s43856-022-00168-7)
Supplement: Supplementary file 4 — Reporting Summary [file 43856_2022_168_MOESM4_ESM.pdf]

nature research

Corresponding author(s): COMMSMED-22-0649-T

Last updated by author(s): May 11, 2022

Reporting Summary

Nature Research wishes to improve the reproducibility of the work that we publish. This form provides structure for consistency and transparency in reporting. For further information on Nature Research policies, see our Editorial Policies and the Editorial Policy Checklist.

Statistics

For all statistical analyses, confirm that the following items are present in the figure legend, table legend, main text, or Methods section.

n/a	Confirmed	
☐	☒	The exact sample size (*n*) for each experimental group/condition, given as a discrete number and unit of measurement
☒	☐	A statement on whether measurements were taken from distinct samples or whether the same sample was measured repeatedly
☒	☐	The statistical test(s) used AND whether they are one- or two-sided<br>*Only common tests should be described solely by name; describe more complex techniques in the Methods section.*
☒	☐	A description of all covariates tested
☒	☐	A description of any assumptions or corrections, such as tests of normality and adjustment for multiple comparisons
☐	☒	A full description of the statistical parameters including central tendency (e.g. means) or other basic estimates (e.g. regression coefficient) AND variation (e.g. standard deviation) or associated estimates of uncertainty (e.g. confidence intervals)
☒	☐	For null hypothesis testing, the test statistic (e.g. *F*, *t*, *r*) with confidence intervals, effect sizes, degrees of freedom and *P* value noted<br>*Give P values as exact values whenever suitable.*
☐	☒	For Bayesian analysis, information on the choice of priors and Markov chain Monte Carlo settings
☒	☐	For hierarchical and complex designs, identification of the appropriate level for tests and full reporting of outcomes
☒	☐	Estimates of effect sizes (e.g. Cohen's *d*, Pearson's *r*), indicating how they were calculated

Our web collection on statistics for biologists contains articles on many of the points above.

Software and code

Policy information about availability of computer code

Data collection	n/a
Data analysis	Genome assembly was performed with Genome Detective Virus Typing Tool v.1.137 available online (https://www.genomedetective.com/app/typingtool/virus). Phylogenetic analyses were performed with Nextstrain Tools (https://nextstrain.org/sars-cov-2/), TempEst v.1.5.3 (https://beast.community/tempest/), BEAST v.1.10.2 (https://beast.community/install_on_mac/), Tracer v1.7.7 (https://tree.bio.ed.ac.uk/software/tracer/), FigTree v.1.4.4 (https://tree.bio.ed.ac.uk/software/figtree/), and iTOL (https://itol.embl.de/).

For manuscripts utilizing custom algorithms or software that are central to the research but not yet described in published literature, software must be made available to editors and reviewers. We strongly encourage code deposition in a community repository (e.g. GitHub). See the Nature Research guidelines for submitting code & software for further information.

Data

Policy information about availability of data

All manuscripts must include a data availability statement. This statement should provide the following information, where applicable:

- Accession codes, unique identifiers, or web links for publicly available datasets
- A list of figures that have associated raw data
- A description of any restrictions on data availability

All genome sequences generated by this study were published in GISAID and a list of accession numbers is available in the Supplementary Table 1 of this manuscript. A complete list of all genome sequences retrieved from GISAID used for phylogenetic context is available in the Supplementary Table 2 of this manuscript and all authors acknowledged.

Field-specific reporting

Please select the one below that is the best fit for your research. If you are not sure, read the appropriate sections before making your selection.

☒ Life sciences ☐ Behavioural & social sciences ☐ Ecological, evolutionary & environmental sciences

For a reference copy of the document with all sections, see nature.com/documents/nr-reporting-summary-flat.pdf

Life sciences study design

All studies must disclose on these points even when the disclosure is negative.

Sample size	A total of 753 SARS-CoV-2 genome sequences were obtained in this study from residual diagnostic samples collected through COVID-19 surveillance led by the Puerto Rico Department of Health and the Sentinel Enhanced Dengue Surveillance System (SEDSS) led by the Centers for Disease Control and Prevention, Dengue Branch. A total of 2,917 genome sequences were retrieved from GISAID to provide phylogenetic context.
Data exclusions	No data was excluded from this study.
Replication	All phylogenetic analyses were performed at least twice and trees were inferred with IQTREE, Nextstrain, and/or BEAST software. All tree topologies were comprable.
Randomization	Selection criteria for sequencing diagnostic samples were as follows: all residual nasal swabs with positive RT-PCR test (CT<28, remaining volume >0.25mL). Selection of genome sequences for phylogenetic analyses was conducted by Nextstrain using metadata from all sequendes published in GISAID at the time of the analysis and randomly selected based on sample collection dates, geographic region of origin and genetic proximity to the focus group sequences (Puerto Rico).
Blinding	Only viruses derived from residual samples were sequenced in this study. PII from the patients was not available to the sequencing/analysis laboratory.

Reporting for specific materials, systems and methods

We require information from authors about some types of materials, experimental systems and methods used in many studies. Here, indicate whether each material, system or method listed is relevant to your study. If you are not sure if a list item applies to your research, read the appropriate section before selecting a response.

Materials & experimental systems

n/a	Involved in the study	
☒	☐	Antibodies
☒	☐	Eukaryotic cell lines
☒	☐	Palaeontology and archaeology
☒	☐	Animals and other organisms
☒	☐	Human research participants
☒	☐	Clinical data
☒	☐	Dual use research of concern

Methods

n/a	Involved in the study	
☒	☐	ChIP-seq
☒	☐	Flow cytometry
☒	☐	MRI-based neuroimaging
